# A stream classification system to explore the physical habitat diversity and anthropogenic impacts in riverscapes of the eastern United States

**DOI:** 10.1371/journal.pone.0198439

**Published:** 2018-06-20

**Authors:** Ryan A. McManamay, Matthew J. Troia, Christopher R. DeRolph, Arlene Olivero Sheldon, Analie R. Barnett, Shih-Chieh Kao, Mark G. Anderson

**Affiliations:** 1 Environmental Sciences Division, Oak Ridge National Laboratory, Oak Ridge, Tennessee, United States of America; 2 The Nature Conservancy, Eastern Conservation Science, Eastern Regional Office, Boston, Massachusetts, United States of America; University of Siena, ITALY

## Abstract

Describing the physical habitat diversity of stream types is important for understanding stream ecosystem complexity, but also prioritizing management of stream ecosystems, especially those that are rare. We developed a stream classification system of six physical habitat layers (size, gradient, hydrology, temperature, valley confinement, and substrate) for approximately 1 million stream reaches within the Eastern United States in order to conduct an inventory of different types of streams and examine stream diversity. Additionally, we compare stream diversity to patterns of anthropogenic disturbances to evaluate associations between stream types and human disturbances, but also to prioritize rare stream types that may lack natural representation in the landscape. Based on combinations of different layers, we estimate there are anywhere from 1,521 to 5,577 different physical types of stream reaches within the Eastern US. By accounting for uncertainty in class membership, these estimates could range from 1,434 to 6,856 stream types. However, 95% of total stream distance is represented by only 30% of the total stream habitat types, which suggests that most stream types are rare. Unfortunately, as much as one third of stream physical diversity within the region has been compromised by anthropogenic disturbances. To provide an example of the stream classification’s utility in management of these ecosystems, we isolated 5% of stream length in the entire region that represented 87% of the total physical diversity of streams to prioritize streams for conservation protection, restoration, and biological monitoring. We suggest that our stream classification framework could be important for exploring the diversity of stream ecosystems and is flexible in that it can be combined with other stream classification frameworks developed at higher resolutions (meso- and micro-habitat scales). Additionally, the exploration of physical diversity helps to estimate the rarity and patchiness of riverscapes over large region and assist in conservation and management.

## Introduction

Classification systems have a long history in stream ecology [1]. Stream classifications serve many fundamental purposes, including understanding similarities and differences among different types of streams, making inferences regarding stream ecosystem behavior, and communicating the complexities of ecosystem function [2]. However, they also provide many applied outcomes, such as grouping sites with similar characteristics [3], stratifying analyses for monitoring and/or experimentation [4], prioritizing aquatic conservation actions [5], and generalizing ecological responses to disturbances [6].

Recent high-resolution geospatial products (e.g., National Hydrography Dataset) have shaped our understanding of stream diversity in the landscape, such as the importance of scale-dependence in understanding stream behavior [7,8] and the importance of dendritic connectivity in organizing aquatic communities [9]. Most contemporary stream classifications, however, have not made use of these latest geospatial datasets. Within the last two decades, stream classifications have either classified groups of discrete observations (e.g., stream gages) [10] or relied on landscape regionalization to deduce behavior of stream environments (e.g., hydrologic landscape regions) [4]. In contrast, the local heterogeneity of riverscapes may be better captured by classifications at the stream-reach scale [11]. Classifications based on stream reaches ensures the scale of classifications match the processes they are meant to reflect. Additionally, these approaches are comprehensive in that they represent all observations, rather than a subset of samples selected purely on the basis of available information [12]. In recent years, the number of studies utilizing stream reach datasets for classification have grown, but applications have been limited to state or sub-regional spatial extents [11, 13–17].

Aside from the issue of spatial resolution, classifications have been catered towards describing natural patterns in individual aspects of physical habitat, primarily hydrology. Hydrologic classifications, as opposed to other measures of physical habitat, have been prioritized because of their utility in informing regional water management and water policy decisions [18,19], as classes represent groups of streams with similar hydrologic characteristics and hence, management units. However, the prevalence of hydrologic classifications is also likely an artifact of the availability of widespread stream gage discharge data [19], whereas other physical measures are either not as numerous or not systematically collected in a standardized way. Less common are classifications for temperature regimes [20, 21] or geomorphological types [16, 22]. Even fewer are classifications that combine multiple habitat features [11, 14, 23]. Incorporating more habitat features in classifications provides a more holistic assessment of stream environments and may also ensure classifications are ecologically relevant [11]. Ultimately, this suggests that contemporary classifications have provided a limited understanding of the regional diversity of stream ecosystems.

At the most basic level, stream classifications are an inventory of different types of streams. Incorporating multiple components of habitat into spatially contiguous stream reach datasets provides an opportunity to inventory the uniqueness of stream environments over large spatial scales. Additionally, examining stream diversity in relation to anthropogenic disturbances provides a gap-analysis framework to prioritize rare stream types lacking natural representation in the landscape [13]. Herein, we estimate the physical habitat diversity of stream environments over the entire Eastern United States (US). To understand the physical diversity of stream habitats, we create a composite classification system of natural patterns in stream hydrology, temperature, stream size, and channel/floodplain geomorphology information within approximately 1 million stream reaches of the Eastern United States. To date, a multi-layered stream classification of this scope or resolution has not been documented in the literature. We focus on the Eastern US as this region encompasses three previous sub-regional classification efforts [23–25] and provides a sufficiently large area to examine heterogeneity in stream physical types. We then compare our typologies to anthropogenic disturbance regimes to examine associations among classes and levels of disturbance. Finally, we overlay the rarity of stream typologies and disturbance levels to prioritize streams for restoration, conservation protection, and biological monitoring [26].

## Methods

### Overview of classification approach

Within aquatic systems, there have been divergent approaches to stream classification, representing varying justifications for classification development [1, 4, 11, 19]. As Melles et al. [1] suggests, there is no single “best” stream classification designed to meet all needs for its use, but to serve as an aid to research, conservation, and management. Our intended purpose for the stream classification was to estimate the diversity of physical habitats in streams within a region. This requires some explanation of terminology. *Layers* represent different categories of stream habitats (e.g. size, temperature) and each layer may have multiple *classes* (e.g., headwater, creek and cold, warm). A *typology* is a specific combination of classes from all layer categories for each stream. By physical diversity, we are referring to the total number of typologies for all streams within a region.

Based on input from advisory board comprised of 41 aquatic ecologists and conservation planners in the Eastern US [23], we selected physical habitat layers that were hypothesized to exert the strongest influence on aquatic habitat structure and the composition of ecological communities and could feasibly be mapped at the stream reach scale. These layers included stream size, gradient, temperature, hydrology, valley confinement, and substrate, in that order of influence; however, we fully admit there are other physical and non-physical factors besides those we considered that are important to stream ecological communities and ecosystem functionality (e.g., chemical properties).

Beyond selecting what information to include in classification lies the difficulty of determining class breaks among observations. Although our layers are relevant to the biological structure of stream communities, we do not partition classes based on biological discriminatory power (see rationale below). In general, we relied on published values of class breaks reported in previous stream classification efforts to distinguish typologies, with emphasis on studies using thresholds defined via expert judgement or via analyses aimed to capture predominant variation in stream types. We excluded studies partitioning classes using ecological community data. Where possible, we attempted to find consensus in the most widely accepted threshold values in the literature. If reported values were unavailable, we relied on clustering to represent predominant physical variation of stream types by capturing breaks in the frequency distributions. We further justify our stream classification approach based on the following criteria:

1. Empirically Based: Inductive approaches to stream classification tend to accurately reflect on-the-ground conditions because utilize information from empirical observations, such as stream discharge gages, in clustering solutions [19]. In contrast, deductive approaches use spatial regionalization or clustering of landscape and climate factors to presume variation in stream diversity [4] andhave been shown to poorly represent actual differences in streams [27].
2. Physical habitat diversity versus biological discrimination: Because classifications are commonly used for conservation management, class partitions are frequently determined by their ability to discriminate amongst biological communities (e.g., [11]). However, there are disadvantages to incorporating ecological information into the classification process. The number of classes in any classification system depends on the source of variation used to partition classes, which changes with scale [28]. Compared to physical properties of streams, ecological datasets are collected within relatively limited spatial and temporal extents [29]; thus, altering the structure of habitat variables to maximize ecological interpretation not only reduces the variation used to determine stream habitat diversity, but also removes the mechanistic linkage between physical process and the structure of ecological dynamics. Hence, we relied on existing classification paradigms using physical patterns (e.g. [22, 30, 31]) to partition classes.
3. Stream reach resolution- Adequately modeling thephysical properties of streams, such as hydrology or temperature, requires assembling information in a fashion that captures upstream processes and the advection of hydrologic forces [8, 32, 33]. This requires a spatial topology among river patches as a template to summarize the cumulative effects of upstream landscape patterns on longitudinally nested river habitats. The NHDPlus dataset [34] provide an ideal framework of topologically connected reaches and serves as an intermediate spatial resolution that scales between watersheds and microhabitats [30, 31, 33].
4. Layered vs Lumped Approach When classifying streams based on multiple habitat layers, there are generally two alternative approaches. Cocktail approaches lump multiple environmental variables into a single agglomeration of classes either deterministically or through multivariate clustering. As an example, Ecoregions represent geographic areas of presumed similarity in climate, hydrology, geomorphology, topography, land use, and ecological communities [35]. In such a deductive classification, one cannot distinguish the diversity of stream systems because individual habitat components have been masked by coarser-scale agglomeration. As another example, Leathwick et al. [11] developed a sophisticated approach to stream-reach scale classification by weighting environmental landscape variables based on their explanatory power of fish and invertebrate distribution data. While the classification was adept at biological discrimination, the physical diversity of individual habitat layers was compromised because cluster solutions collectively grouped stream properties based on minimizing within-group variation.
  In contrast, layered approaches compartmentalize streams into different components of habitat or functional properties (e.g., [36]). Additionally, layered classifications make no pre-defined judgement of the relative importance of different habitat constituents [14], and as such, they are flexible and can accommodate for the selection of one or many components that may be relevant to capturing stream physical diversity, depending on the application. While we recognize that different habitat components are interrelated and hierarchically structured, using the relative importance of habitat components in structuring ecological communities or processes to govern estimates of physical diversity of stream systems is problematic because any measure of relative importance is completely scale-dependent [14, 27].
5. Natural conditions–Understanding the physical diversity of stream systems, especially how stream diversity has been impacted by anthropogenic disturbances, requires physical habitat layers be based on natural (i.e., minimal disturbance) conditions. In this case, classes might represent idealized habitat conditions to guide restoration and management. If habitat layers within stream classifications are based on natural conditions, then altered conditions can always be added as an additional layer as the reference provides the baseline to measure the altered condition.

### Layer development

Given the properties mentioned above, we developed a physically-based layered classification of stream reaches based on empirically-driven observations of six natural habitat characteristics including: hydrology, temperature, size, gradient, valley confinement, and substrate. We use the stream network topology of the NHDPlus version 1, 1:100,000 scale (NHDPlus V1) as the spatial template of our classification. The spatial extent includes hydrologic regions 1 through 6 of the Eastern US [37]. Within this region, there are approximately 1.5 million km of stream reaches; however, classifying all of these reaches was not possible because many streams lack geospatial variables for predicting habitat values. Most of these streams are braided or consist of artificial channels with no topological connection to other reaches, which prevents network routing to accumulate geospatial information. Many spatially-explicit datasets (e.g., climate, soils, geology, landforms, baseflow index, contact time, etc.) were downloaded, processed, and attributed to all NHDPlus v1 catchments in the Eastern US in a Geographic Information System. Almost 200 variables were derived from these datasets and summarized for each NHDPlus v1 catchment (S1 File). These variables were then accumulated for the entire drainage network upstream of each stream reach using the NHDPlus v1 Catchment Attribute and Accumulation Tool (CA3T) with the total upstream drainage routing option [34]. Variables were then used to develop models to predict stream class membership.

Size, gradient, and confinement layers relied on stream attributes derived directly from remote sensing information for all stream reaches; thus, observations for these layers were comprehensive and represented the entirety of stream geography in the region. However, appropriately characterizing hydrology, temperature, and substrate layers required us to summarize data from empirical field observations and then construct predictive models to extrapolate those patterns to all stream reaches in the landscape. Because the field observations represented only a subset of streams, we conducted an analysis to determine whether streams including our observation datasets were representative of the overall variation of streams within the Eastern US (SI File). We found that the subset of streams containing each of our observational datasets (hydrology, temperature, and substrate), capture over 98.5% of the overall variation represented by the entire stream dataset (S2 File). Hence, we felt that our empirical observations sufficiently covered a diversity of streams in the region.

#### Size

Stream size is indicative of major ecosystem changes along the river continuum, such as transitions in the state and source of energy to ecosystem metabolism [38]. The earliest approaches to stream classification differentiated stream size based on order [39, 40]. While this seems like a straightforward approach to size classification, stream order is influenced by the scale of mapped hydrography and may not be indicative of river discharge [41]. Alternatively, mean annual discharge provides a continuous metric of size and is proximally-linked to ecological processes [42]. Although mean annual discharge estimates are available from NHD [34], we elected to use upstream drainage area because this metric has been most commonly applied in most recent stream classification systems [23, 43], is more versatile as it represents both the scale of upstream landscape processes and network position [44] and is more readily available in many regions of the world relative to flow information. Additionally, drainage area correlates strongly with mean annual discharge and the correlation remains consistent across all regions in our study area (S3 File). Cumulative upstream drainage area (km^2^) is provided as an attribute with the NHDPlus V1 dataset. We used a combination of literature review of physically-based stream size thresholds [13, 24, 25, 30, 43; 44; 45] and k-means clustering to explore potential breaks in size classes (S1 Fig). We log(x+1) transformed drainage area and then partitioned size into different clusters using a similar k-means clustering approach as described above (from 2 to 15 cluster solutions). We found consistency among values reported in literature (S1 Fig), as well as congruence among our class breaks and those of the previous size classifications; thus, we relied on these published values to determine our class breaks (Table 1, S1 Fig).

**Table 1:** Value ranges used to partition size, gradient, temperature, and substrate classes. Codes for classes are provided.

Size Classes	Range (km^2^)	Gradient Classes	Range (Rise/Run)	Temperature Classes	Range (°C)	Substrate Classes	Range (mm)
Headwater (HW)	0–10	Very Low (VL)	< 0.001	Cold (CD)	12–19	Sand- Fine Gravel (SG)	0–16
Creek (CK)	10–100	Low (L)	0.001–0.005	Cold-Cool (CC)	19–21	Coarse Gravel (CG)	16–64
Small River (SR)	100–500	Moderate (M)	0.005–0.02	Cool (CL)	21–23	Small Cobble (SC)	64–150
Medium River (MR)	500–2500	Moderate High (MH)	0.02–0.04	Cool-Warm (CW)	23–25	Large Cobble (LC)	150–300
Mainstem (MS)	2500–10000	High (H)	0.04–0.1	Warm (W)	25–30	Small Boulder (SB)	300–600
Large River (LR)	10000–25000	Steep (S)	> 0.1	…..	…..	Large Boulder (LB)	600–1000
Great River (GR)	> 25000	…..	……	…..	…..	Large Boulder-Bedrock (LBB)	1000–1604

#### Gradient

Gradient (i.e., stream bed slope) exerts control on stream physical habitats, including channel morphology, hydraulics, sediment transport, habitat units, and substrate [46]. Rosgen [22] provides a geomorphic classification of rivers based on stream channel typologies describing predominant variation in channel dimensions, sinuosity, entrenchment, and substrate grain size across the US. Despite the documented criticisms and limitations [47], Rosgen’s classification is useful for research [47], has been widely institutionalized by management agencies [48], and has been previously used for partitioning gradient classes within other stream classification efforts [24, 43]. Rosgen [22] provides six major gradient thresholds (Table 1), which we used to portion stream reaches into gradient classes based on stream slope values obtained from flowline attributes within the NHDPlus V1 dataset. Stream slope values were determined using smoothed elevations created from 30-m digital elevation model data. The difference in maximum and minimum values (i.e., rise) were summarized along each NHDPlus flowline and divided by the total length of the flowline (i.e., run) [34]. It should be noted that NHDPlus flowline length underestimates true sinuosity and total length of stream reaches; thus, gradient is slightly over-estimated in most cases.

#### Hydrology

The natural flow regime of rivers, measured as the magnitude, frequency, duration, timing, and rate of change of flow creates and maintains habitat [49], organizes aquatic organism life histories [50], and sustains the structure and function of aquatic and riparian ecosystems [51]. Numerous examples of hydrologic classifications exist where long-term stream discharge records are statistically summarized and clustered using computationally demanding algorithms, resulting in groups of streams with similar hydrologic behavior (for a review, see [19]). A recent hydrologic classification was completed for the entire U.S. using US Geological Survey (USGS) stream gages representing reference-quality hydrologic information [52]. Stream gages were clustered into 15 different types using a Bayesian mixed modeling technique based on hydrologic statistics summarizing at least 15 years of discharge data. These classes represent natural variation in hydrology and classes are not influenced by river size (by standardized magnitude-related statistics). Within the Eastern US, 897 of these USGS gages represented 9 of the 15 hydrologic classes (Table 2). We developed a random forest model to predict hydrologic class membership based on 192 variables including landscape, climate, topography, and soil variables summarized in drainage basins above each stream gage (S1 File). Using this model, we then extrapolated hydrologic class membership to all NHD stream reaches in the region.

**Table 2:** Criteria defining differences in hydrology and confinement class membership. Hydrology class membership and descriptions were based on [37].

Classes	Description/Criteria
**Hydrologic Classes**	
Intermittent Flashy 2 (IF2)	High intermittency
Late Timing Runoff (LTR)	Semi-stable, late annual maximum due to hurricanes
Perennial Runoff 1 (PR1)	Seasonally variable and semi-stable, but lower baseflows, higher variability, and different timing than PR2
Perennial Runoff 2 (PR2)	Seasonally variable and semi-stable, but higher stability, higher baseflows than PR1
Snowmelt 2 (SNM2)	Distinct and consolidated seasonal periods of runoff, stable, relatively high baseflows
Stable High Baseflow (SHBF)	High baseflows, stable, and relatively high runoff
Super-stable Groundwater (SSGW)	Very high baseflows, very high stability, but not necessarily high runoff
Perennial Flashy (PF)	Moderate intermittency, low predictability, semi-flashy
**Confinement Classes**	
Unconfined (UC)	Valley bottom covering > 50% stream reach length and valley bottom width > 4X bankfull width
Moderately Confined (MC)	Valley bottom covers 25%-50% of stream reach length and valley bottom width > 4X bankfull width. Or valley bottom covers > 50%, but valley width is 2–4 X bankfull width.
Confined (C)	All reaches not falling into either of the categories below

#### Temperature

Stream temperature imposes physiological limits on freshwater organisms [53], while exerting controls on ecosystem metabolism and nutrient processing [54]. Recent efforts to examine natural variability in stream temperatures suggest that summer temperatures (July-August averages) are the most influential determinants of divergent thermal regimes [21, 55, 56]. We compiled temperature data from multiple sources, including 762 USGS gauges with daily records [56] and 1588 individually deployed temperature loggers from federal and state agency personnel [55]. Of these, 869 sites were determined to be representative of reference conditions based on the National Fish Habitat Risk Assessment (NFHA) [57] and evaluating the degree of upstream regulation (DOR) by impoundments (see disturbance section for definition). Reference conditions were determined as sites having NFHA assessment scores as “low” or “very low” and DOR < 4% [58]. Using the same predictor ensemble for hydrology, we developed random forests to predict summer temperatures for the reference sites and then extrapolated those values to all NHD stream reaches (S1 File). Using estimated summer-time temperature values for all stream reaches in the region, we used a k-means clustering procedure to partition classes. To determine the most parsimonious solution that explained the most variation, sum-of-squared-distances (SSD) within groups were compared to number of clusters (from 2 to 15 cluster solutions). We selected the smallest number of clusters than minimized the SSD within group. The resultant class threshold values are provided in Table 1.

#### Valley confinement

Valley confinement refers to the potential of river channels to migrate and interact with their floodplain. The degree of valley confinement determines the formation of floodplains and riparian wetlands and the interaction between channel morphology and floods, which influence sediment budgets and formation and maintenance of in-stream and riparian habitats [59, 60, 61]. A similar metric is entrenchment, technically defined as a ratio of floodplain width to bankfull width, where the floodplain is measured at 2X bankfull height [22]. We used the Valley Confinement Algorithm (VCA) tool [62] within ArcMap 10.2 to delineate unconstrained valley bottoms for all NHDPlus stream reaches. VCA estimates bankfull depth of the stream channel based on regional precipitation data and drainage area for each stream reach [63]. Flood height is determined based on a user-defined flood-factor multiplied by bankfull depth. Nagel et al. [62] recommends 5X bankfull depth is appropriate for defining valley bottoms. Based on 30-m DEMs, the program uses an algorithm to intersect flood height with the surrounding hillslope to general floodplain valleys as polygons. Once valley bottoms are delineated, thresholds are required to determine whether valley bottoms extend laterally beyond the bankfull width a sufficient distance to be constitute an ‘unconstrained’ stream reach. This required estimating bankfull width for each stream reach. We used a combination of i*n situ* field observation and remote sensing information from 1708 sites to develop an empirical model to predict bankfull width for all stream reaches. Field observations of bankfull width (n = 1403) were derived from Environmental Protection Agency’s (EPA) National Rivers and Streams Assessment (NRSA) [64]. However, the EPA NRSA assessment tended to exclude headwater and very large systems. Thus, we randomly selected a subset of NHDplus stream reaches (n = 305), stratified by stream size, in the Eastern US to augment the sample of bankfull width measurements. We used aerial imagery to estimate bankfull width for stream reaches at the midpoint, upstream, and downstream end of each reach (if visible) and then calculate mean bankfull width. Random forest models were used to predict bankfull width and extrapolate estimates to all stream reaches using 156 predictors (S1 File). Bankfull width estimates were then used to generate polygon buffers around all streamlines.

Bankfull widths were then compared to valley bottoms to determine valley constraint status. Typically, stream channels with entrenchment ratios of > = 4 are considered unconfined [63] whereas streams with entrenchment ratios between 2 and 4 indicate some moderate interaction between channels and their floodplain [22]; however, these estimates only consider a single point and not the extent of the floodplain in relation to the entire stream reach. Thus, it’s difficult to arbitrarily classify reaches as either “confined” or “unconfined” without some intermediate category. Based on the values above, we classified stream reaches as “unconfined if the valley bottom had widths at least 4X that of the bankfull width and covered at least 50% of the stream reach length. Moderately confined stream reaches met the following criteria: 1) valley bottoms had widths ≥ 4X the bankfull width, but only covered 25%–50% of the stream reach length, or 2) valley bottoms with widths 2-4X the bankfull width and covered 50% of the stream length. All other stream reaches were defined as confined.

#### Substrate

Stream substrate type and size exerts a strong influence on invertebrate community composition [65], variant life history strategies for fish [66], and the nature of periphyton colonization [67]. We compiled 1059 field measurements of streambed surficial substrate particle sizes from stream habitat assessments from the EPA NRSA (n = 586) and EPA National Stream Survey (NSS) (n = 473) databases [68]. Of these, 459 sites (NRSA, 243; NSS, 216) were representative of reference conditions using the same protocol as temperature monitoring stations. At each field site, multiple transects were laid perpendicular to the channel and multiple grids were established per transect [69]. In each grid, dominant substrate size was determined visually or manually based on the Wentworth scale [70]. We then calculated the percentage of grids at each site falling into different substrate size categories, which included %fines (0.03mm), % sand (1mm), %gravel (33mm), %big rock (1055mm—cobble through boulder sizes), and %bedrock (2056mm). Based on percentages with each substrate size category, we estimated a weighted mean diameter of substrate at each site (this is not analogous to D_50_). Using 159 predictors, we developed random forests to predict mean substrate diameter for each site and then extrapolated those values to all NHDPlus V1 stream reaches (S1 File). The Wentworth scale of grain-sizes [70] is a widely adopted template for classifying substrate size categories; thus, we defined substrate classes based on mean weighted substrate diameter using a modified Wentworth scale (Table 1). Given that mean weighted substrate diameters were biased towards larger particles, we combined finer Wentworth categories (sand and fine gravels, coarse gravels) and expanded the cobble and boulder Wentworth classes into separate types (small and large cobble, small and large boulder, large boulder-bedrock complexes) (Table 1).

### Anthropogenic disturbance

As described earlier, layered approaches provide the ability to consider both the natural and altered condition of streams. We considered four measures of disturbances in stream environments: landcover (urbanization or agriculture), impoundment and dam regulation, upstream fragmentation imposed by dams, and a cumulative measure of disturbance. For urbanization and agriculture, landcover types were summarized as the percentage of upstream cumulative area under all developed landcover types and crop or pasture landcover types, respectively. Using spatial coverages of NHDPlus waterbodies, we identified all stream reaches impounded by reservoirs. Dam regulation was assessed for each stream reach using Degree of Regulation (DOR), a measure of the percentage of annual runoff volume stored within all impoundments upstream [58, 71]. For the DOR calculation, dam locations and storage values were obtained from the National Anthropogenic Barrier dataset [72] and summarized within each stream reach’s upstream network. For each stream reach, fragmentation was assessed in two ways: 1) a binary indication of whether a reach was connected (i.e., unobstructed flow) to the ocean, and 2) a Dendritic Connectivity Index (DCI), which is a percentage of a reach’s upstream functional network distance relative to total upstream network distance [9]. The functional network is calculated as the total distance of stream environment available upstream bounded by dams. A cumulative disturbance index was developed by the National Fish Habitat Partnership (NFHP), as a multi-metric indication of landscape-related risks to fish habitats in US streams [57, 73]. The index summarizes many anthropogenic disturbances (e.g., urbanization, number of dams, pollution discharge permits) for the entire network upstream of each reach in the US. However, we observed that the cumulative disturbance index only includes the number of upstream dams and does not account for the magnitude of dam regulation (i.e., dam storage) or fragmentation (i.e., length of streams fragmented by dams). Thus, we assigned stream reaches to classes of hypothesized disturbance levels from high-to-low intensities: 1) impoundment, 2) noticeable dam regulation or DOR > = 4% [58], 3) fragmented upstream watershed or DCI < 75%, or 4) landscape alteration if the NFHP cumulative disturbance index fell under “moderate”, “high”, or “very high” values. If any of the conditions above were met, a stream reach was considered “disturbed”. Otherwise, a stream was classified as “not disturbed”.

### Class-disturbance associations

Because some habitat layers may show a greater prevalence to disturbance, we evaluated associations between classes for each layer and disturbance levels. We modeled associations between physical habitat layers on each continuous disturbance variable mentioned above using generalized linear models [74]. All binary or proportional disturbance variables were modeled as binomial distributions whereas DOR was modeled as a gamma distribution following log(x+2) transformation and scaling all values to >1. Associations among each physical type and disturbance classes were evaluated using *X*^*2*^ tests and measures of association strength (Phi-Coefficient and Cramer’s V).

### Biological sampling

Of practical importance is understanding how well the diversity of physical types have been biologically surveyed. Almost 900,000 occurrence records of fish, mussels, and crayfish were compiled using several open-access databases for the entire US and overlain with NHDPlus V1 catchments [29]. Records were included if they documented the year, latitude, and longitude of collection. All records collected prior to 1990 were removed from consideration as these are not representative of present-day surveys. Records were summarized as binary indicators of occurrence for fish, mussel, crayfish, or a combination of the above. The frequency of stream reaches biologically sampled was compared among different physical stream types.

### Stream habitat diversity and uncertainty

#### Diversity

In the previous sections, we evaluated each physical habitat layers separately; however, stream typologies represent combinations of habitat layers. Obviously, various combinations of habitat layers will yield different numbers of stream typologies. An example of a combination of habitat layers (Table 1) could resemble the following: CK-M-PR2-CD-UC-LB, which represents a Creek with moderate gradient, perennial runoff 2 hydrology, cold temperatures, unconfined valley geomorphology, and large boulder substrate. Evaluating the diversity of stream habitat types required that we first identify a relevant number of layer combinations to consider. However, the degree to which new typologies emerge with additional combinations of layers can indicate the degree of inter-relationships among layers. For instance, a high-gradient channel is also very likely to be confined. In situations of high inter-relationships among layers, increasing combinations of habitat layers will yield little increases in unique typologies, i.e. minor additions to diversity. In this case, we refer to the addition of these layers as redundant. Assuming layers are completely independent of one another, a complete factorial combination of habitat layers can be used to estimate the number of expected typologies ($\hat{C}$):

$$\hat{C}=\prod_{i}^{n}K_{i}$$

Where *K* represents the number of classes within the *i*^*th*^ habitat layer (e.g., temperature, gradient) and *n* represents the number of habitat layers being considered. In this case, all possible class combinations for all habitat layers (*n* = 6) yields 39,690 expected classes. Because of inter-relationships among layers, $\hat{C}$ is likely to greatly exceed the observed combination of classes (*C*). Based on these values, we can calculate a diversity score as an indication of redundancy, by dividing *C* by $\hat{C}$.

To balance redundancy with information gained from including more layers, we compared the diversity score to different combinations of physical layers. Combinations included all layers together, various combinations of 5 layers, and combinations of 4 layers. An optimal number of layer combinations seeks to maximize the diversity index while simultaneously minimizing losses in information (i.e., increasing numbers of classes). Thus, an optimum occurs where the diversity score reduces towards an asymptote with increasing numbers of classes. Once an optimum number of physical type combinations was identified, we calculated the rarity of each class as the cumulative length (km) represented in the landscape.

Some of the geospatial data (e.g., PRISM climate) used to derive variables in stream reaches were relatively coarse (e.g., 800m) and could influence the predicted classification for small streams. We determined that classes with cumulative lengths < 1km could be an artifact of underlying data rather than a legitimate stream class. Thus, we separated classes with cumulative lengths > 1km for all subsequent analyses.

#### Uncertainty

Mapping stream classes in the landscape does not come without uncertainty, and this uncertainty can propagate through combinations of classes used to develop stream typologies, thereby influencing our diversity estimates. We compiled estimates of deviations in layer values (e.g., temperature, °C) or estimates of probabilities in class membership, in the case of hydrology, to develop a range of possible values for each stream reach (S4 File). Based on class partition thresholds (Table 1), we then re-classified all stream reaches for each layer. If partition tresholds were exceeded, we assigned each reach to 2^nd^ most probable class (S4 File). We then developed a series of new stream typology scenarios by varying classes within each layer independently and then varying all layers collectively. The number, cumulative stream length, and rarity (defined below) of new typologies were compared to the optimal number of typologies defined above (i.e., simple typologies). On a reach-by-reach basis, we also examined the agreement between the original simple typology and all typology scenarios. We measured agreement as the percentages of stream length sharing similar typologies and sharing similar rarity.

### Prioritization scenarios for conservation protection, restoration, and biological monitoring

A physical inventory of stream reaches provides an opportunity to assess the physical uniqueness of stream types and prioritize streams for different objectives. We developed three prioritization scenarios for streams in the Eastern US with divergent objectives: conservation protection, restoration, and biological monitoring. Prioritizing streams for conservation protection aims to identify streams that are rare and have little or no human disturbance. Using the optimal number of physical combinations (previous section), we ranked stream types from highest to lowest prevalence (according to total length) and generated a cumulative frequency distribution. We selected rare classes as those with total lengths falling within the lowest 10^th^ percentile of all classes. From these classes, we selected stream reaches classified as “Not disturbed”.

Restoration prioritization also aims to identify rare classes, but in contrast to conservation protection, it identifies streams with high disturbance levels. Additionally, streams having biological information can inform and assess the effectiveness of restoration practice. We developed a disturbance threshold (*D*), which simultaneously considers both the prevalence of a stream type from the cumulative frequency distribution and the disturbance frequency of a class (i.e., the proportion of stream length classified as disturbed). *D* was estimated using the following inverse logistic equation:

$$D=L+\left(\frac{d}{1+e^{s*(X_{r}-X_{0})}}\right)$$

Where *L* represents the lower limit of acceptable disturbance frequency for the rarest stream type and *d* represents the maximum limit of acceptable disturbance frequency for the most abundant stream type, *S* is the steepness of the decline in *D*, *X*_*r*_ is class rank with higher values indicating higher abundance, and *X*_*0*_ represents the class rank identified as the point of inflection. It should be noted that *d* is an additive component that takes *L* into consideration, i.e. maximum disturbance is *d* + *L*. Additionally, because class rank directly relates to cumulative frequency, X_0_ can be identified on the basis of cumulative frequency thresholds.

Conservation planning goals have often used protected-area targets of 10% per biome [75]; however, these have been shown to under-represent biomes and species in jeopardy [76]. Rodrigues et al. [77] showed that countries with at least 60% protected areas had virtually no gap species (species whose distribution does not overlap protected areas). Presuming the loss of rare stream types has a greater likelihood of jeopardizing species declines, we adopted a conservative goal of restoring at least 60% of stream length within highly rare stream types and at least 10% of stream length of dominant stream types. Concurrently, we determined that the rarest and most abundant stream types should have no more than 40% and 90% of stream mileage being classified as disturbed, respectively; thus, *L* = 0.4 and *d* = 0.5. *S* was assigned a value of -0.0125 because it provided a desirable steepness to avoid over-inclusion of abundant stream types as values approached critical cumulative frequency thresholds. Out of 1983 total stream classes, the 284 most abundant classes represented 90% of total stream length. Hence, *X*_*0*_ was assigned a value of 1700 as this represents the 90^th^ percentile critical disturbance threshold and an appropriate inflection point. All stream types with disturbance frequencies surpassing D were selected. From these, we identified individual stream reaches that fell within one of the disturbance categories (e.g., impounded, fragmented) and had been biological sampled for fish, crayfish, or mussels.

While identifying where biological information exists is important, prioritizing streams lacking biological monitoring is equally important, as this constitutes an information gap [13, 26]. This is especially true of rare streams facing high risk of anthropogenic disturbance with biological communities in jeopardy of homogenization or sensitive species loss. We selected streams whose class disturbance frequencies > D and whose entire class had not been biologically sampled since 1990.

## Results

Out of 1.5 million km of stream reaches in the Eastern US, approximately 125,000 km of streams (8.3%) were unavailable to classification due to inability to summarize geospatial variables in stream networks (i.e., braided or artificial channels). The remaining 1.38 million km of stream reaches (91.7%) had sufficient geospatial characteristics to assign physical classes deterministically or through predictive modeling.

**Fig 1:**
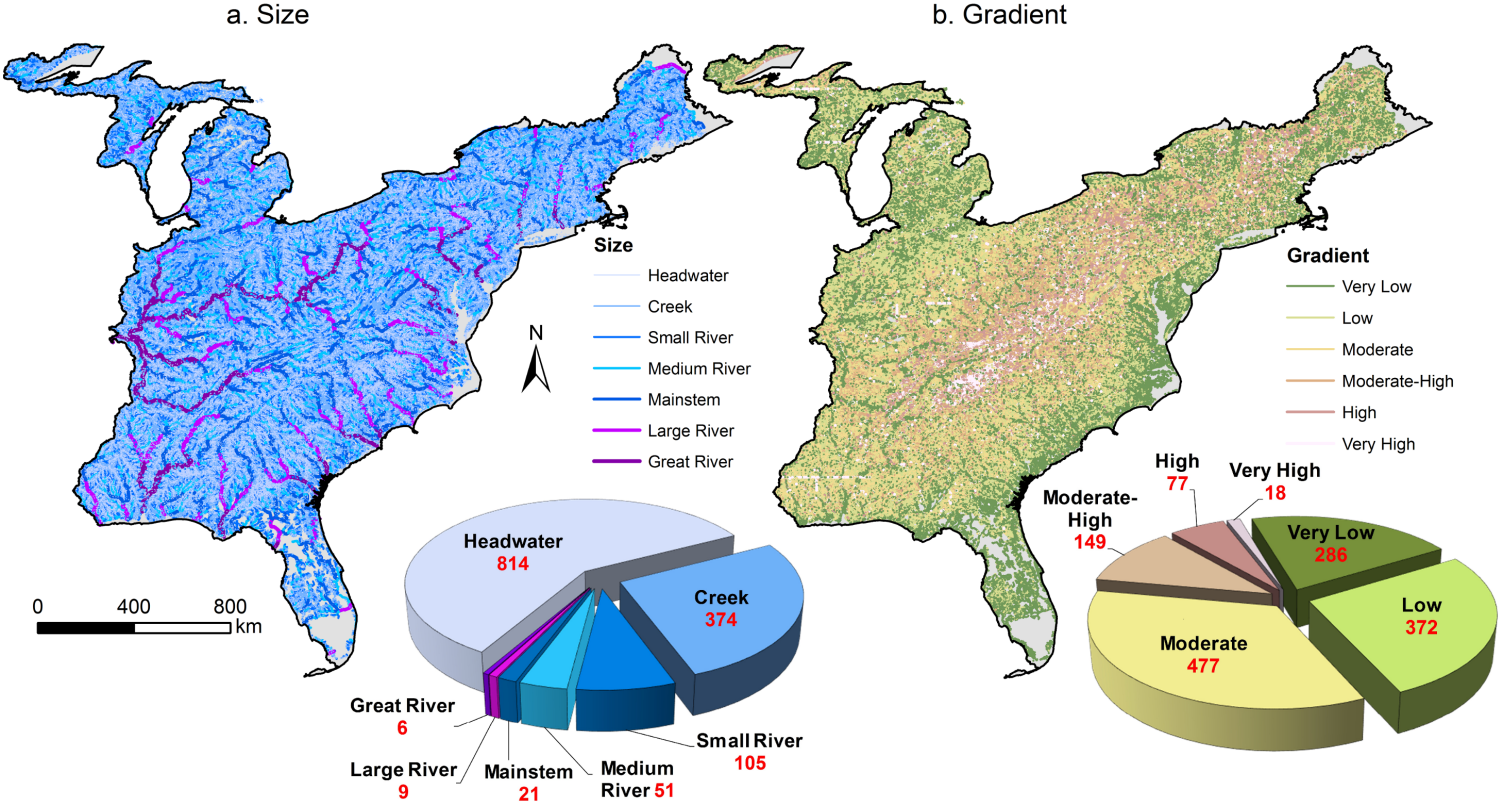
The distribution of size and gradient classes. (a) Size and (b) gradient classes for stream reaches based on drainage area and channel slope, respectively. Total length of streams per class reported as 10^3^ km.

Based on literature values, we identified seven size classes based on upstream drainage area (Table 1). Stream lengths among size classes displayed an exponential decay distribution, with small systems having the highest frequency and very few large river systems (Fig 1a). Headwater streams and creeks comprised 54% and 25% of total stream length, respectively. Approximately, 1% of streams were classified as Large or Great Rivers (≥ 10,000 km^2^). Gradient breaks were based upon values provided by Rosgen [22]. Streams were dominated by moderate (32%), low (25%), and very-low gradient (19%) classes (Fig 1b).

**Fig 2:**
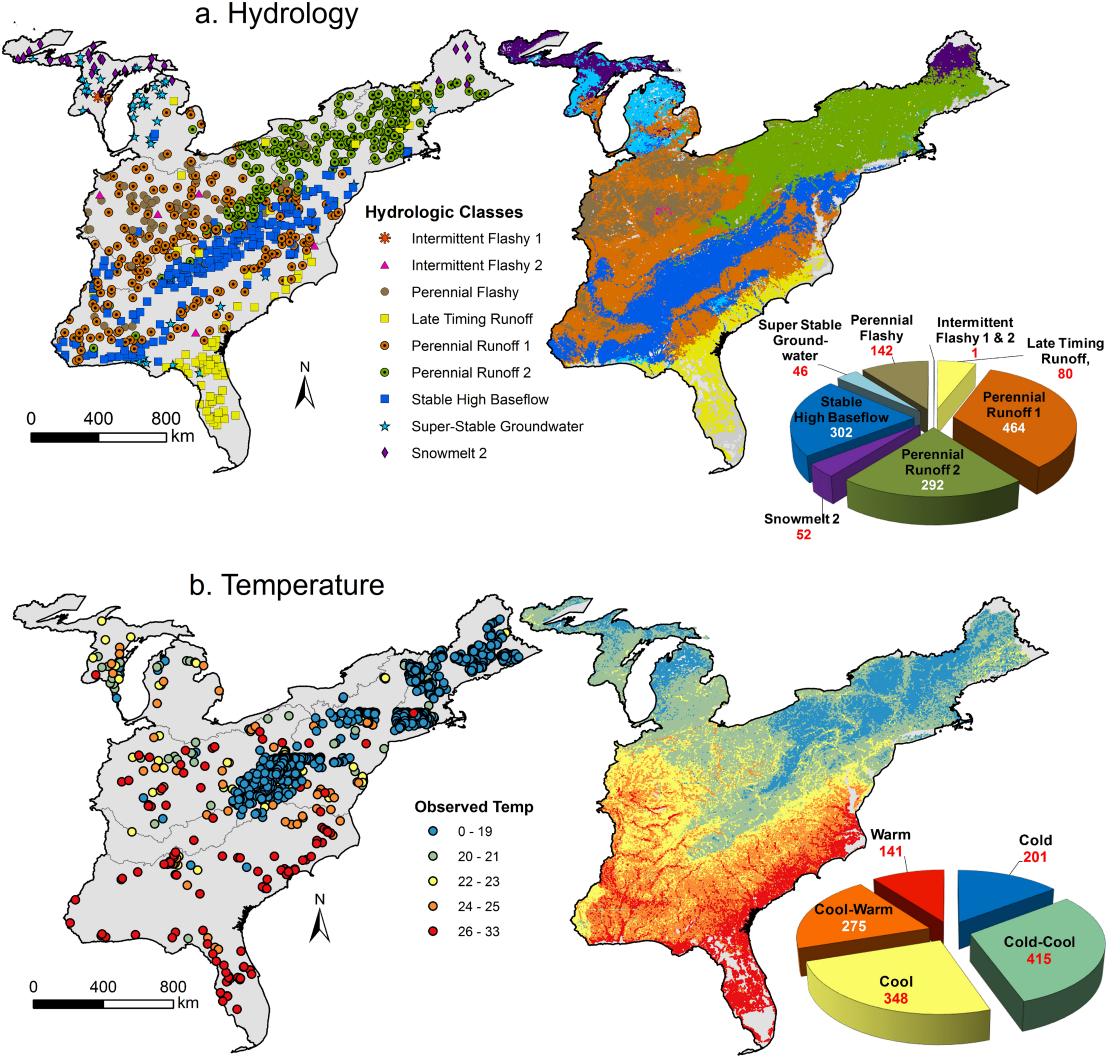
The distribution of hydrologic and temperature classes. (a) Hydrologic classes from stream gages [37] were mapped to stream reaches using predictive models. (b) Temperature classes developed from average July and August stream temperatures at monitoring stations extrapolated to stream reaches. Total length of streams per class reported as 10^3^ km.

Random forests predicting hydrologic classes had 80.7% out-of-bag accuracy (cross-validation rate). Variables characterizing hydrology (e.g., baseflow index) and climate had the highest relative importance in models, followed by soil characteristics (S1 File). Hydrologic classes represented by fewer stream gauges typically had higher error rates (S1 File). For example, intermittent flashy 1 and 2 types were extremely rare (< 4 observations), which resulted in 100% misclassification error for those hydrologic types. Error rates for other classes ranged from 0.08 to 0.55. The most abundant hydrologic types were Perennial Runoff 1 and 2 streams and Stable High Baseflow streams (Fig 2a).

Summer-time average temperature varied across the Eastern US (Fig 2b). Random forests predicting mean summer temperatures explained 70.5% of variation in the out-of-bag sample (S1 File). For the K-means analysis, sum-squared distances within groups minimized at five cluster solutions (Table 1). Cold-Cool (28%) and Cool (23%) streams were the most abundant whereas Warm (9.4%) streams were the rarest (Fig 2b).

**Fig 3:**
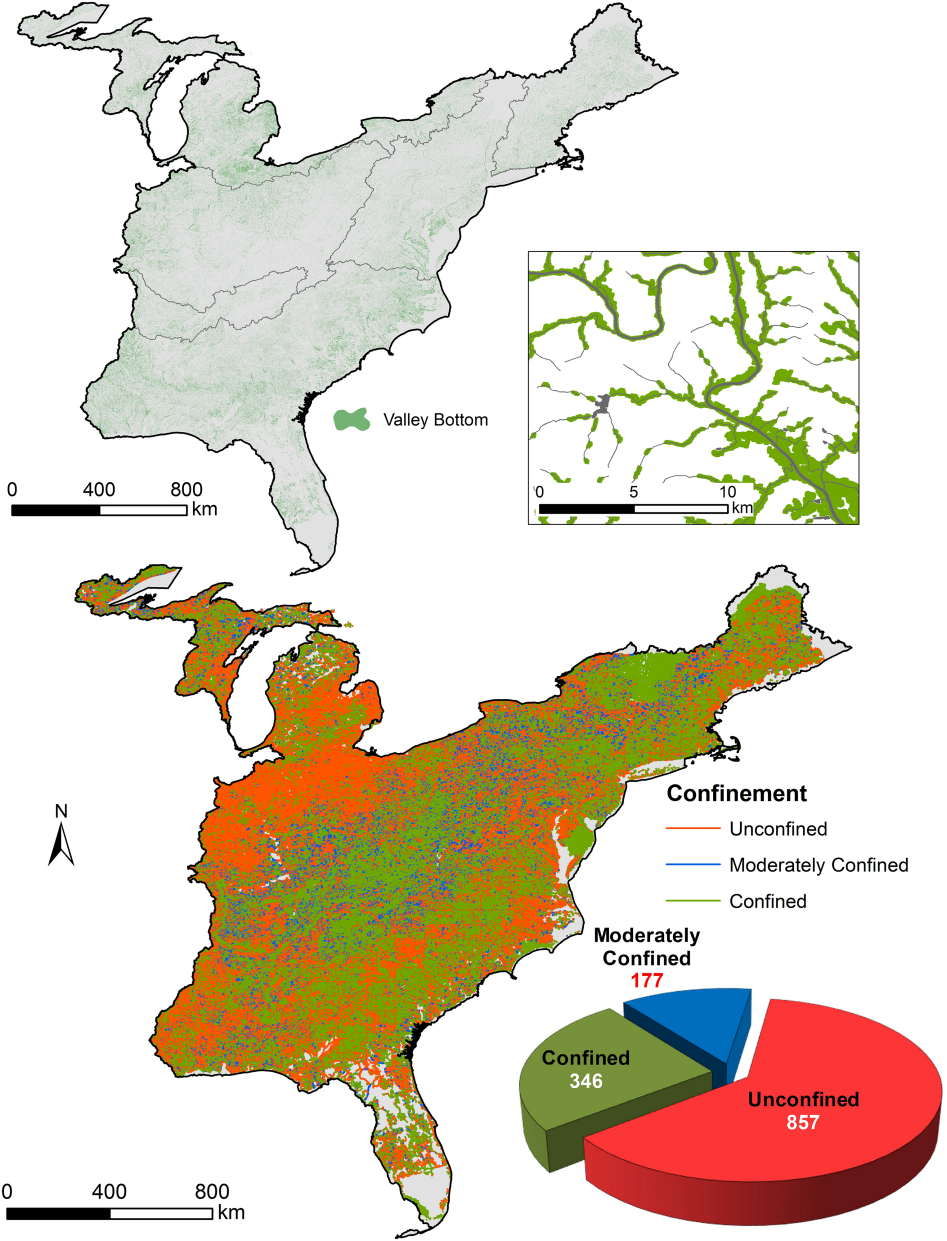
The distribution of valley confinement classes. Valley bottoms delineated for stream reaches and used to determine confinement class. Example close-up of valley bottom and stream network. Total length of streams per class reported as 10^3^ km.

Within the Eastern US, the VCA tool generated over 1.2 million valley bottoms with a cumulative area approximating 219,000 km^2^ (Fig 3). Random forests explained 84.5% variation in bankfull width (S1 File). The majority of stream reaches (56%) were classified as unconfined compared to moderately confined (12%) and confined (23%) (Fig 3).

**Fig 4:**
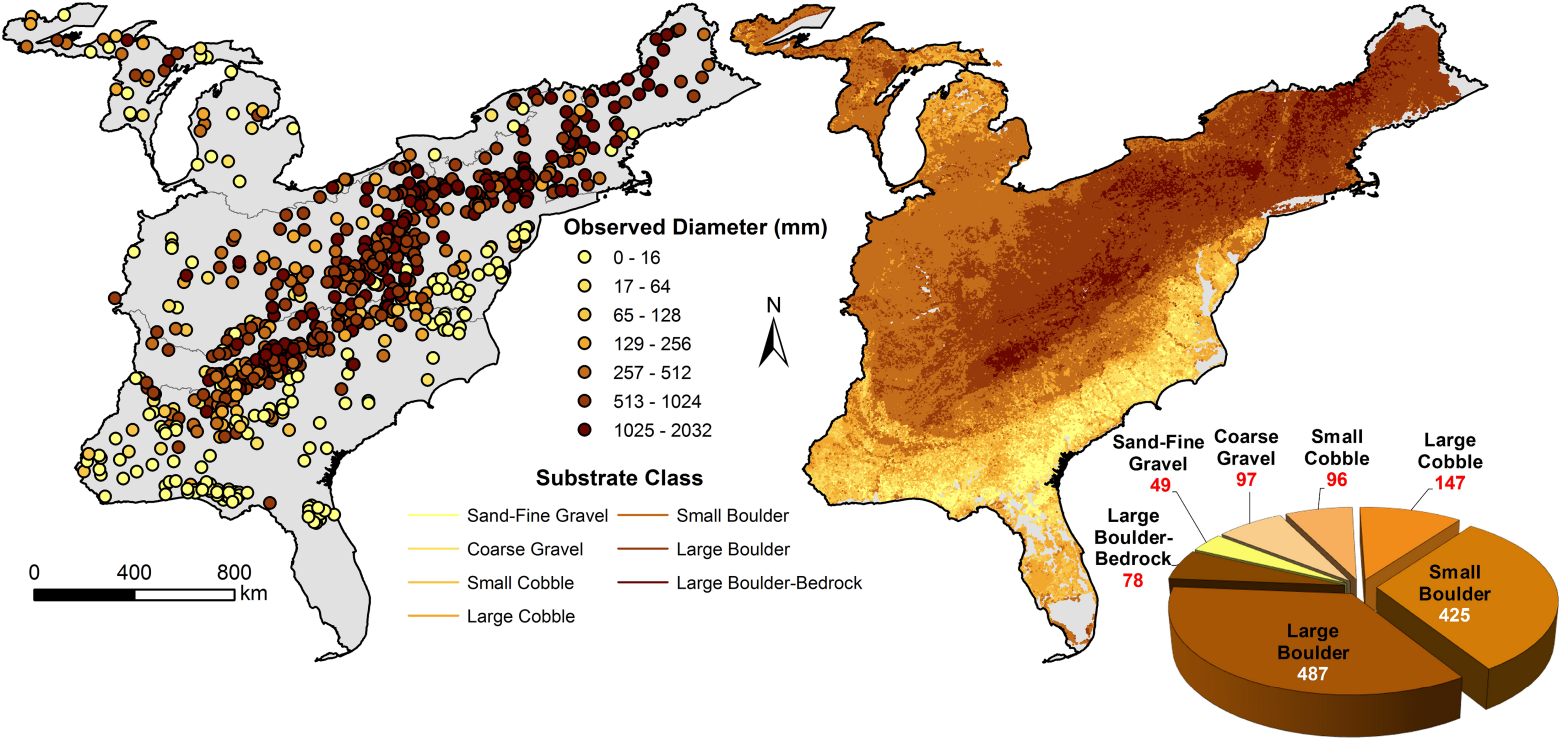
Substrate classes developed from weighted mean particle sizes at field locations and extrapolated to stream reaches. (median particle size not available). Total length of streams per class reported as 10^3^ km.

Random forest models explained 56.8% of variation in mean substrate diameters (S1 File). Based on a modified Wentworth scale (Table 1), we portioned substrate diameters into seven substrate classes (Fig 4). Almost 61% of streams were dominated by small boulder or large boulder stream types (Fig 4).

### Stream habitat diversity and uncertainty

#### Diversity

**Fig 5:**
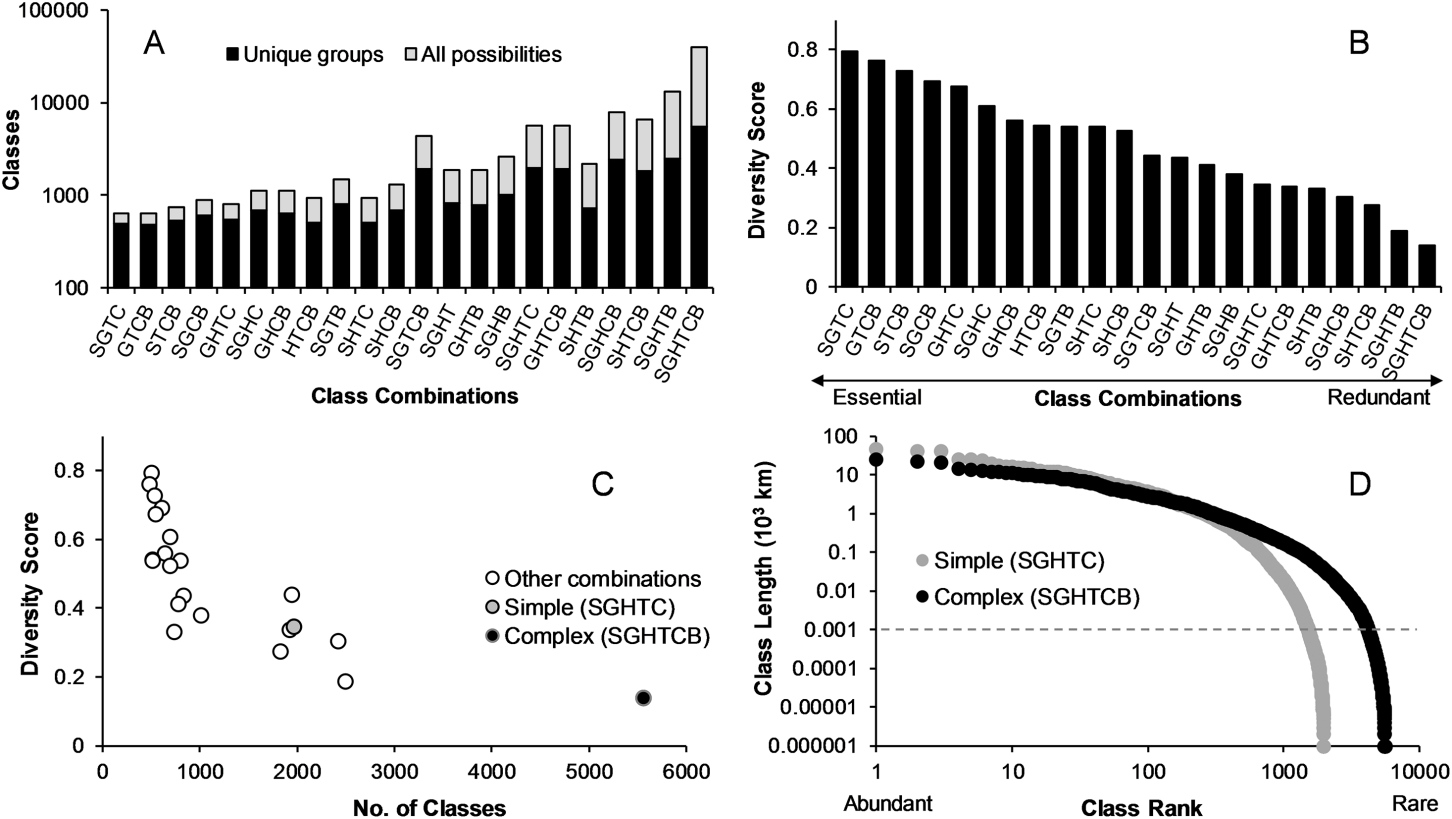
An assessment of stream class diversity, redundancy, and uniqueness. (A) Various combinations of stream classification layers yield observed (i.e., unique) and theoretical (i.e., potential) numbers of classes. (B) Diversity score (observed/theoretical classes) relative to various combinations of stream class layers. (C) The optimal number of classes is found where the diversity score minimizes. This attempts to strike a balance between removing redundancy and losing information on stream physical diversity. Complex classes refer to all combinations of stream layers (n = 5,577 classes) whereas simple classes refer to all combinations minus the substrate layer (n = 1983). (D) Distribution of class lengths relative to abundance. Simple classes tend to shift the distribution into fewer, more abundance classes that have cumulative lengths > 1km.

When considering all six layers, the total number of combined stream classes was 5577 types compared to the 39,690 potential types (Fig 5a) (For a subset of typologies, see S1 Table; All typologies listed in S5 File;). Hence, the diversity score is very low (0.14) and suggests there is a great deal of redundancy relative to the expected diversity. Approximately, 25% of classes (n = 1389) were represented by < 1km of total stream length and potentially an artifact of uncertainty in predictor information. Various combinations of 4 and 5 different physical types yielded a range of diversity scores from highly essential to highly redundant combinations (Fig 5b). A combination of size, gradient, temperature, and confinement had the highest diversity score (Fig 5b). When plotted against the number of classes, the diversity score minimized at an intermediate combination of 5 physical stream class types representing a balance between only essential classes and potential information lost (Fig 5c). Because of the poor predictive accuracy of modeling substrate size in stream reaches and the fact that substrate classes did not contribute greatly to uniqueness, we simplified the classification by excluding substrate and retaining the other five layers (size, gradient, hydrology, temperature, and confinement), representing 1983 typologies, 1521 of which were represented by at least 1 km (S5 File). Of these, 305 classes made up 90% of the total length of streams in the Eastern US, and 430 classes made up 95% of the total length (Fig 5d).

#### Uncertainty

By imposing uncertainty in classes, we developed a series of typology scenarios (S4 File). The majority of scenarios resulted in diversity and rarity estimates similar to that of the original simple typologies (Table 3). When considering uncertainty in all six layers, the total number of combined classes was 6856. After excluding substrate, our typology estimates ranged from 1966 to 2140 when we only varied individual layers. Varying all layers collectively, however, resulted in 2679 total typologies (Table 3). Excluding stream typologies < 1km resulted in 1434 to 1650 typologies for varying individual layers and 2027 when all layers were allowed to vary. The number and percentage of rare types was similar between the original simple typology and that of all scenarios (Table 3).

**Table 3:** Comparison of the total and rare typologies emerging from the simple typology versus those arising from scenarios assessing uncertainty in class membership. Rare types refer to stream typologies with cumulative lengths in the lowest 10^th^ percentile of all typologies. Layers do not consider substrate classes.

Typology Scenarios	Total Types	Total Types >1km	Rare Types >1km	% Rare Types >1km
Simple (Original)	1983	1521	1217	80.0
Variant Size Class	1974	1514	1210	79.9
Variant Gradient	1987	1529	1220	79.8
Variant Hydrology	2140	1650	1216	73.7
Variant Temperature	1872	1434	988	68.9
Variant Confinement	1966	1506	1126	74.8
All Layers Varied	2679	2027	1501	74.1

On a reach-by-reach basis, variant size, gradient, and confinement classes had 96%, 79%, and 97% agreement, respectively, with the original simple typologies (S4 File). Variant hydrology and temperature classes had 42% and 34% agreement, respectively. When all layers were varied, new typologies only had 12% agreement with original simplified typologies. Agreement in patterns of rarity were generally higher than that of all streams (S4 File). Variant size, gradient, and confinement classes had 98%, 87%, and 65% agreement, respectively, with the original simplified typologies (S4 File). Variant hydrology and temperature classes had 45% and 39% agreement, whereas varying all layers had 31% agreement.

### Disturbance

**Fig 6:**
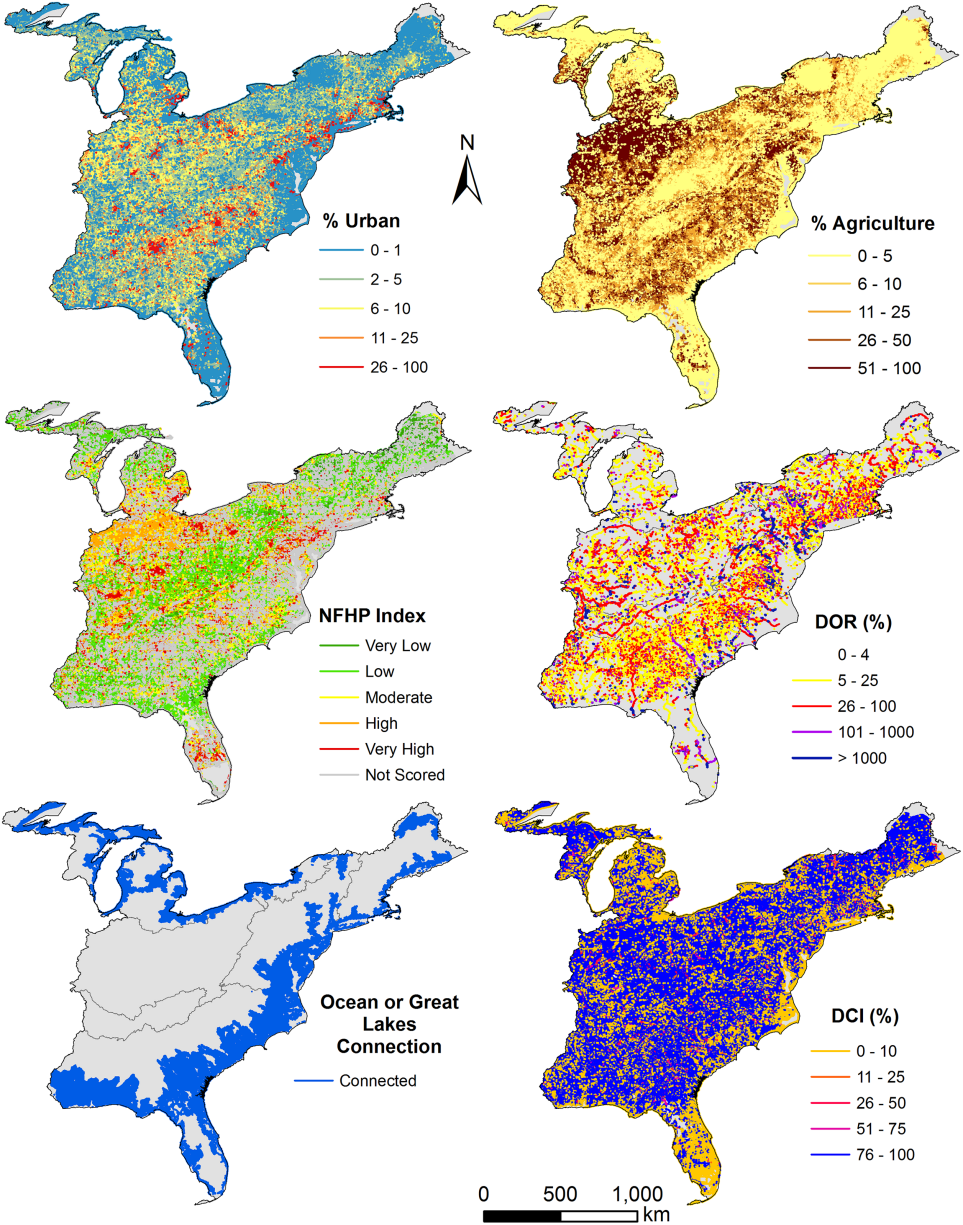
Disturbance regimes mapped to stream reaches.

**Fig 7:**
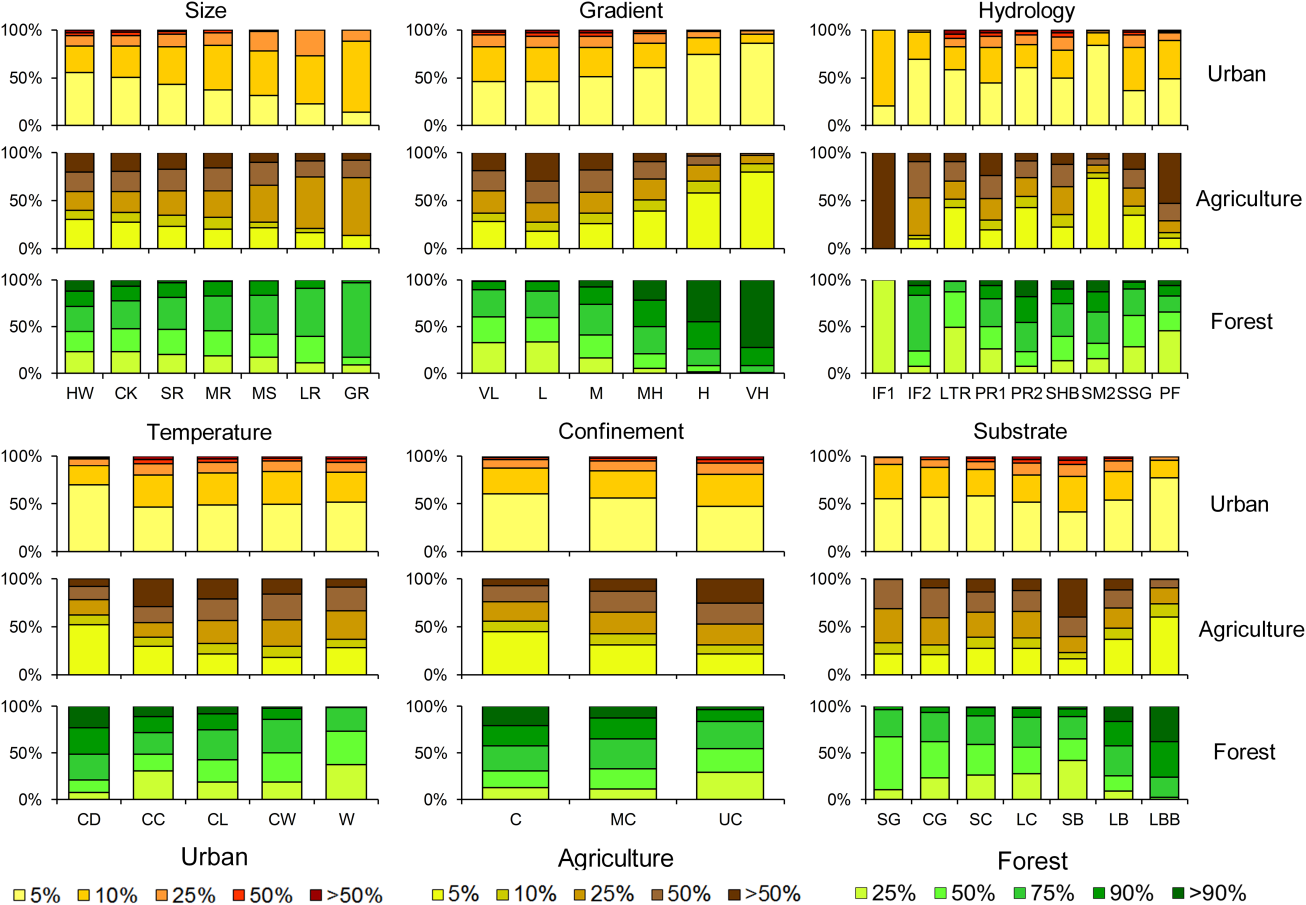
Proportion of stream length having various levels of % agriculture or % urban land cover in their upstream watersheds according to stream classes.

**Fig 8:**
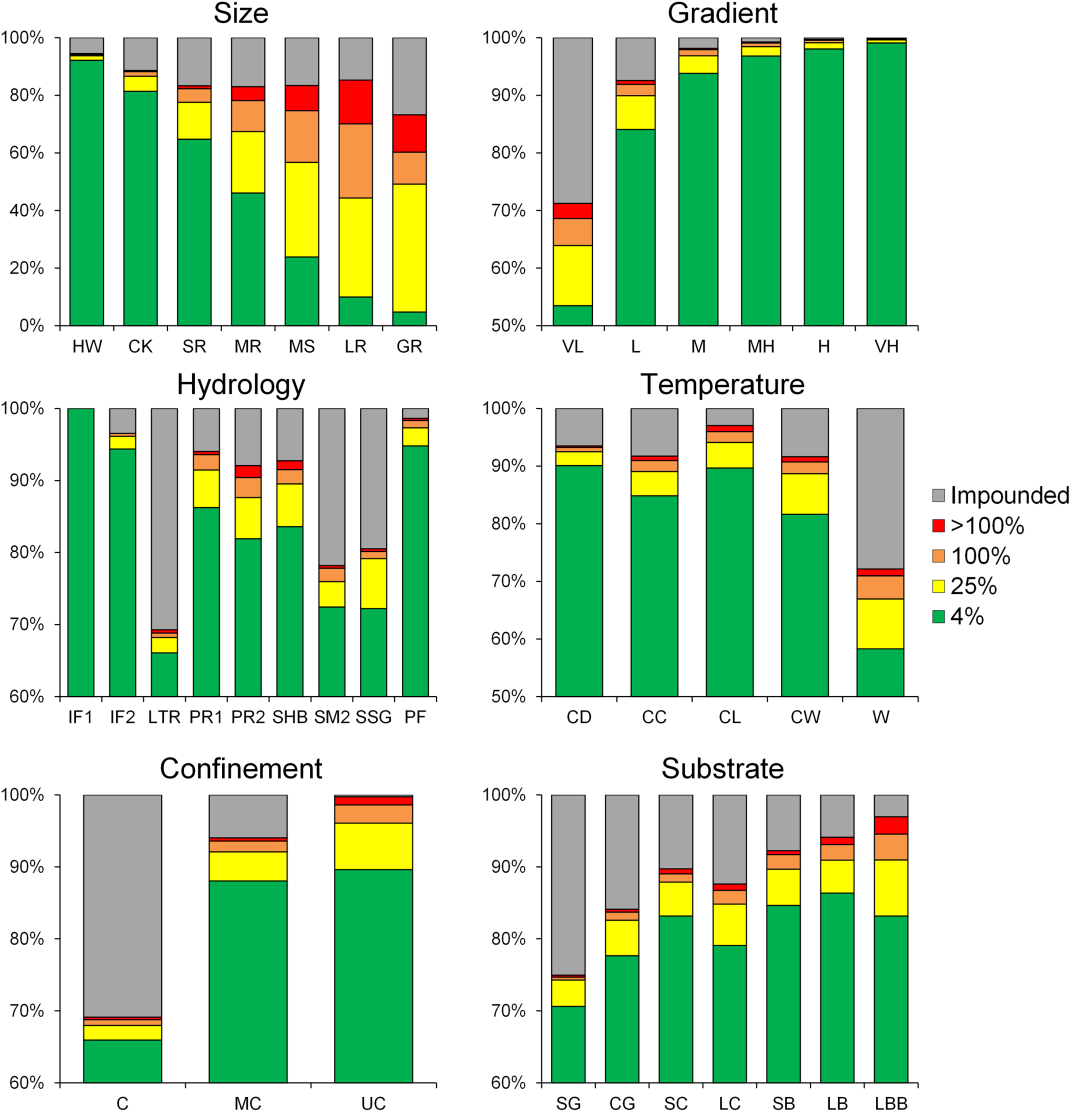
Proportion of stream length either impounded or regulated by various degrees by impoundments according to stream classes.

**Fig 9:**
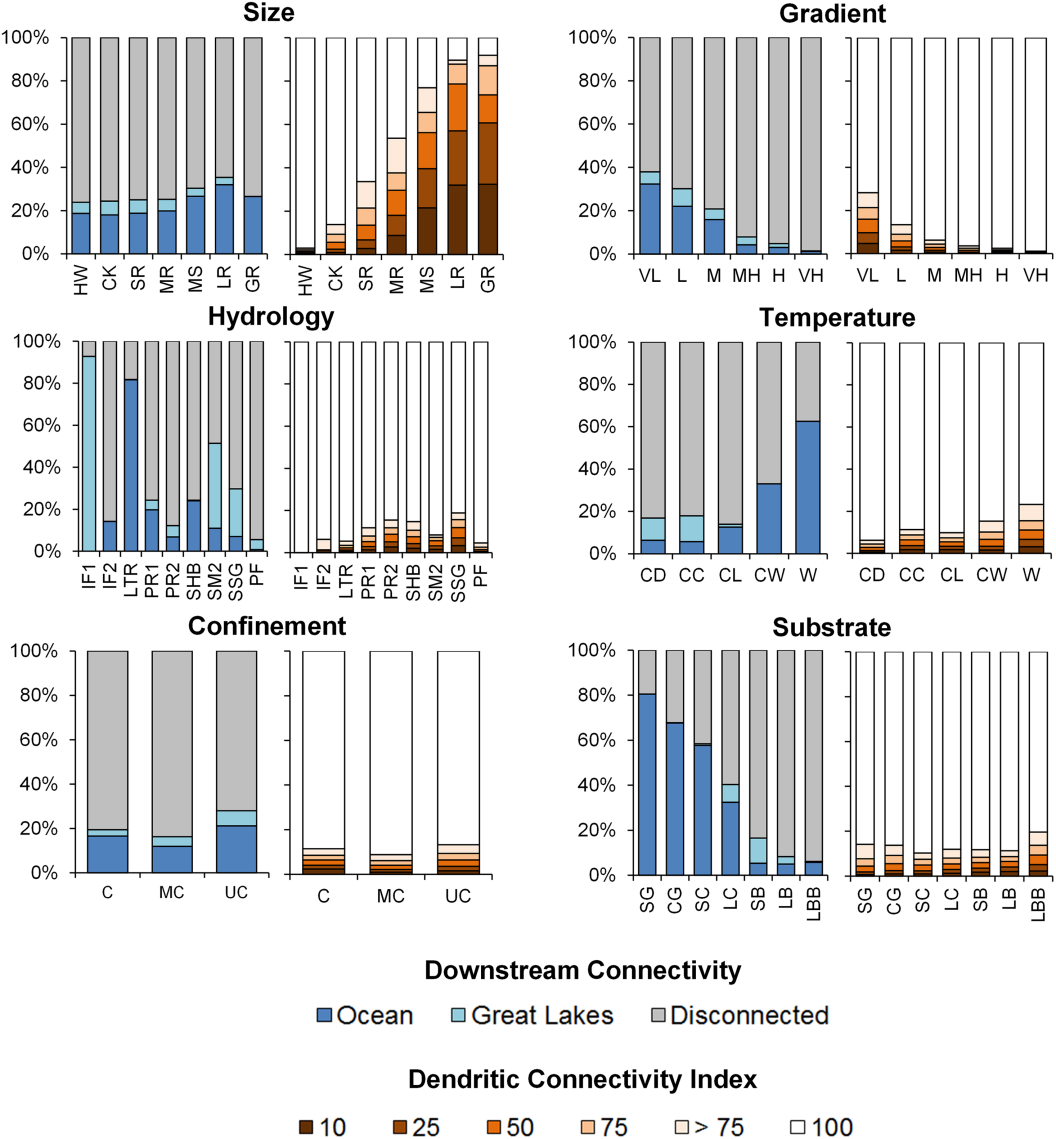
Proportion of stream lengths according to stream class membership connected (free-flowing) to the ocean or a Great Lake and falling under various levels of fragmentation, as measured by the Dendritic Connectivity Index (see [9]).

Landscape disturbance, dam regulation, and dam fragmentation was widespread across the Eastern US (Fig 6). Approximately 16% of streams have at least 10% urbanized land cover in their upstream networks whereas 5.4% have at least 25% upstream urbanization (Fig 7). Agricultural impacts were more pervasive with 56% and 37% of streams having at least 10% and 25% agriculture land coverage, respectively, in their upstream network (Fig 7). Most streams (90%) have some upstream regulation by dams (DOR > = 1%); however, only 7.2% of streams have DOR > = 4% (Fig 8). Additionally, 9.5% of stream reaches are impounded (Fig 8). Almost 78% of streams are disconnected from their terminus in an estuary or Great Lake. Only 17% of streams are connected to the Atlantic Ocean or Gulf of Mexico whereas 5.1% of streams are connected to a Great Lake (Fig 9). Despite the degree of disconnection to estuarine or Great Lakes, almost 81% of streams have fully connected upstream watersheds (i.e., DCI = 100%) (Fig 9). Considering overall disturbances, 69.5% of streams in the Eastern US are either impounded, regulated, highly fragmented, or have significant landscape disturbances.

### Class-disturbance associations

The strength of associations between disturbances and stream classes, as measured by Generalized Linear Models, varied considerably. Relationships were strongest for models predicting % agriculture and DCI from stream classes (Table 4). Except for size, all stream classes explained ≥ 78% of the variation in % agriculture (Nagelkerke R^2^) whereas size, gradient, and hydrology explained ≥ 74% of the variation in DCI (Table 4). Relationships between stream classes and other disturbance variables were weaker than those for DCI and % agriculture. Confinement and gradient classes explained 54% and 16% of variation in impoundment rates, respectively (Table 4). Size was the only class explaining noticeable variation (25%) in DOR. Gradient, hydrology, temperature, and substrate classes explained 20% to 36% of variation in urbanization and 9.2% to 32% of the variation in connectivity (Table 4). All classes had significant associations (p < 0.0001) with overall disturbance regimes (Table 5). Confinement classes had the strongest association with overall disturbance, followed by size and gradient classes (Table 5).

**Table 4:** Nagelkerke’s R2 values and McFadden’s pseudo-R^2^ (for DOR) from generalized linear models predicting disturbances from stream classes.

Disturbance	Size	Gradient	Hydrology	Temperature	Confinement	Substrate
Impoundment^1^	0.002	0.159	0.061	0.059	0.540	0.034
DOR^2^	0.245	0.101	0.033	0.015	0.007	0.013
Connectivity^1^	0.000	0.092	0.212	0.156	0.008	0.323
DCI^1^	1.000	0.890	0.742	0.378	0.131	0.234
Urbanization^1^	0.021	0.220	0.236	0.199	0.044	0.360
Agriculture^1^	0.011	0.863	0.989	0.781	0.808	0.978

Modeling using ^1^binomial distribution or ^2^gamma inverse distribution

**Table 5:** Measures of association between stream classes and disturbance classes. Disturbance classes are a categorical indication of the anthropogenic stressor, if present, inducing the largest influence on a stream and includes (in order of greatest to least influence): Impoundment, dam regulation, dam fragmentation, landscape alteration, and no disturbance.

Disturbance	Df	Likelihood ratio	X^2^	Phi	Contingency	Cramer’s V
Size	24	139346	188596	0.47	0.426	0.235
Gradient	20	154060	159111	0.432	0.397	0.216
Hydrology	32	63407	64714	0.276	0.266	0.138
Temperature	16	61665	64966	0.276	0.266	0.138
Confinement	8	348589	321971	0.615	0.524	0.435
Substrate	24	55599	56277	0.257	0.249	0.128

### Prioritization scenarios for conservation protection, restoration, and biological monitoring

**Fig 10:**
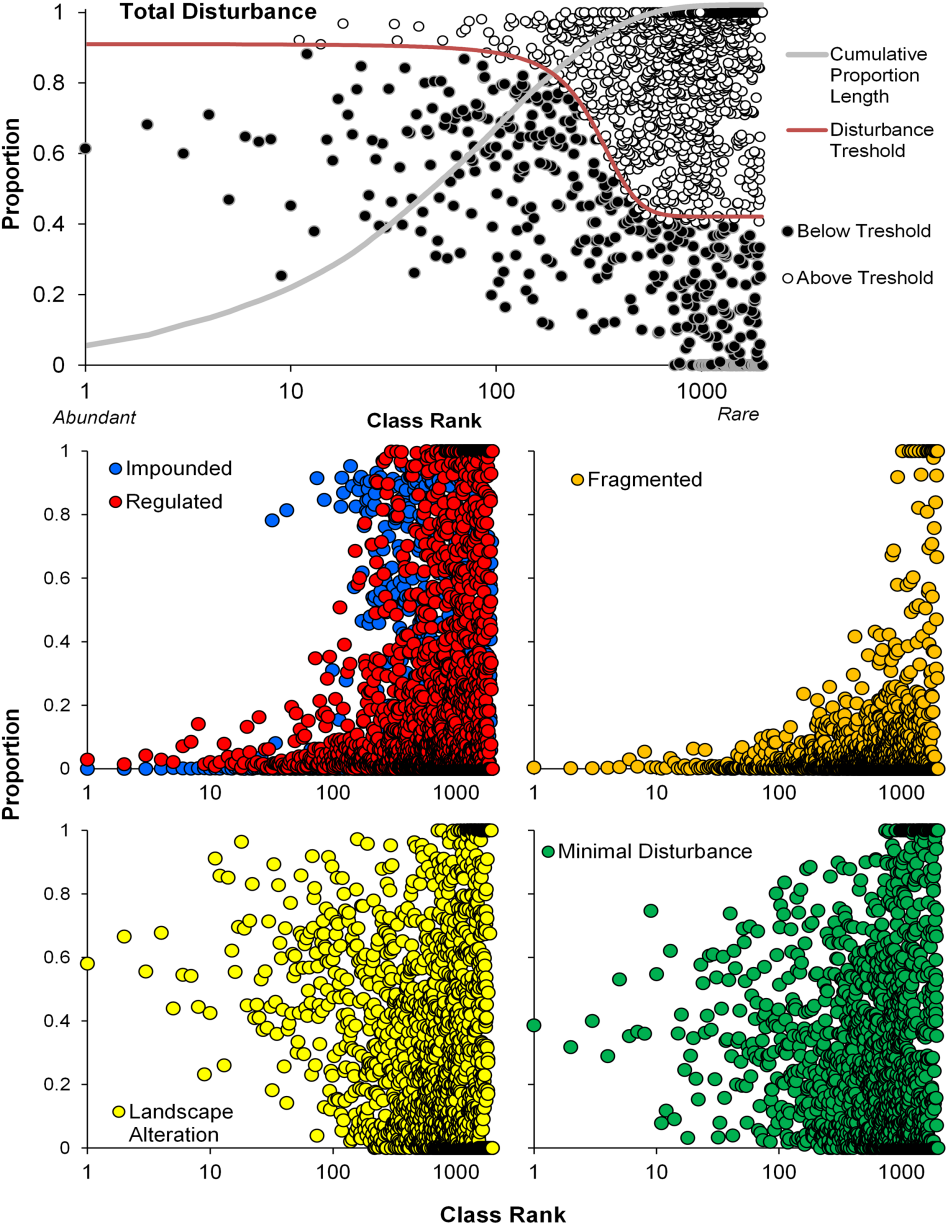
Simple stream classes whose rarity and disturbance levels exceed the disturbance threshold. Proportions of stream length within each simple class are plotted against class abundance ranking. The cumulative frequency of stream lengths versus class abundance is provided. The inflection point of the disturbance threshold occurs at the 90^th^ percentile of class abundance. Proportions of class membership (based on length) that fall under individual disturbance regimes are compared to class abundance ranking.

**Fig 11:**
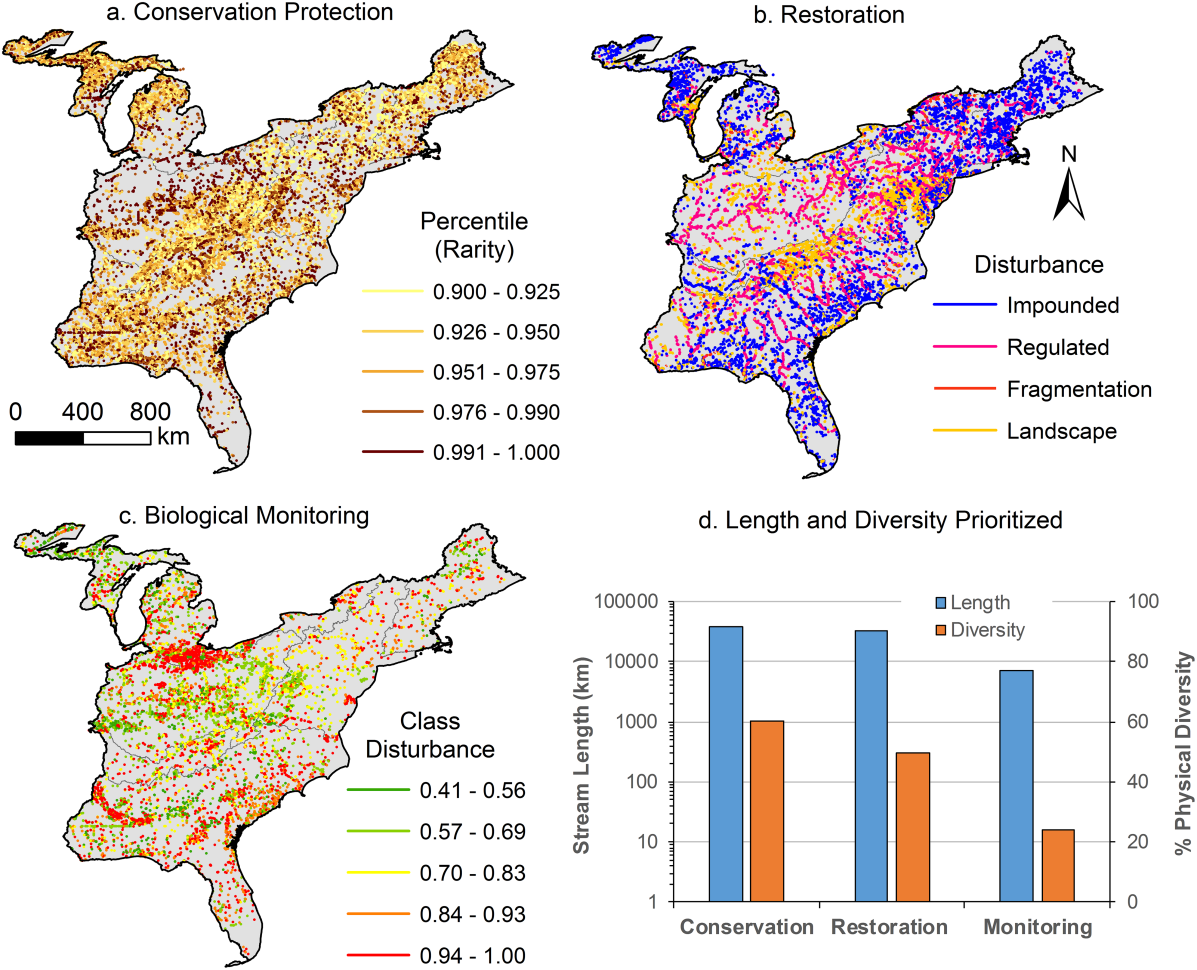
Prioritization of streams based on (a) conservation protection where rare streams with cumulative lengths in the top 10^th^ percentile (highest percentile are the most rare) also have no known anthropogenic disturbances, (b) restoration for stream reaches with class disturbance levels exceeding the disturbance threshold and individually falling within one of the four disturbance classes, and (c) biological monitoring where streams with class disturbance levels exceeding the disturbance threshold, and in classes not biological sampled since 1990.

Of the 1521 simple typologies, a total of 306 classes have 100% of their streams categorized as disturbed, yet represent 20% of the physical diversity of streams in the Eastern US (Table 6, Fig 10). Additionally, 492 classes (32%) have at least 95% of their stream lengths classified as disturbed. A total of 36 classes have been completely lost to impoundment. Classes with disturbance frequencies exceeding D totaled 1151, which represented 75% of the physical diversity in the entire region and 24% of the total stream length (Table 6, Fig 10). We identified 916 classes for conservation protection, which represented 60% of the physical diversity of streams in the Eastern US, but only 2.5% of the overall stream length for the region (Table 6, Fig 11). Of the classes with disturbance frequencies exceeding D, we selected 752 classes for restoration, which represented 49% of the diversity in the region, but only 2.1% of overall stream length. Finally, streams prioritized for biological monitoring represented 363 classes, 24% of the regional diversity, but < 0.5% of the overall length (Table 6, Fig 11).

**Table 6:** Filtering criteria for prioritizing streams according to rarity and disturbance regimes. D refers to Disturbance Threshold.

Filters	Classes	Length (km)	Reaches	% physical diversity	% stream length
Class Disturbance > D	1151	361592	279548	75.7	24.0
Class Disturbance = 100%	306	136521	141038	20.1	9.07
Class Disturbance > 95% Disturbance	492	250888	256866	32.3	16.7
Classes with 100% Impoundment	36	252	163	2.37	0.02
Prioritization					
Conservation Protection	916	38047	22540	60.2	2.53
Restoration	752	32186	13319	49.4	2.14
Biological Monitoring	363	7134	7397	23.9	0.47
All Prioritizations	1328	77367	43256	87.3	5.14

The patterns in disturbance and diversity remained similar even when we imposed uncertainty in classes and typologies (S2 Table). Thus, the prioritization scenarios were only conducted for the original simplified typologies.

## Discussion

Understanding the diversity of streams across large spatial extents highlights commonalities and uniqueness in stream ecosystems [78]. Heterogeneity in stream physical diversity provides a spatial template [79] or landscape filter [80] to examine the influence of physiochemical variation and anthropogenic disturbance regimes on ecological strategies. These patterns are of practical significance because they can be used to assess stream rarity and prioritize conservation efforts. Additionally, generalizing streams into classes has relevance to increasing the representation of finer-scale stream processes in large-scale models of Earth’s ecosystems [81].

Within the Eastern United States, we estimate there are anywhere from 1,521 to 5,577 different physical types of streams based on combinations of our habitat layers. However, if we consider uncertainty in mapping classes to stream reaches, those estimates could range from 1,434 to 6,856 different types of streams. Out of 1521 simplified types, the most dominant stream typology is a headwater, moderate-gradient, stable-high baseflow, cool, unconfined stream, which comprises over 47,500 km (3%) of stream length in the Eastern US (S1 Table). In comparison, the rarest simplified type is a small-river, high-gradient, stable-high-baseflow, cool-warm, confined stream, which comprises only 1 km. The range in potential physical typologies should be expected as this represents a balance between capturing all dimensions of physical rarity while removing redundancy arising from interrelated physical layers. Indeed, stream classification requires striking a balance between preserving the “[individuality] of every stream” [82] with generalizations of stream ecosystem properties. Realistically, streams represent a continuum of physical values in multiple dimensions. While this creates a challenge for classification approaches that seek arbitrary boundaries among typologies, streams are predisposed to classification because their physical attributes display repeatable patterns in the landscape [78]. Additionally, we consider the estimates of physical types as conservative. For instance, our estimates of the physical diversity of stream types would have increased considerably had we included smaller headwater and ephemeral systems.

The majority of stream length (95%) in the Eastern US is represented by less than 30% of the total diversity of stream types (430 of 1521 classes). This suggests that most stream types are numerically rare and non-redundant in the landscape. Additionally, we find that 32% of the physical diversity of stream types have been virtually lost to anthropogenic disturbances. Of these, 36 classes (2.5% of stream diversity) are completely impounded and are unlikely to be restored to a natural condition. Even when we accounted for uncertainty in stream typologies, patterns of rarity and disturbance were consistent and, in some cases, higher that simplified typologies (S2 Table). While stream classes with no natural representation represent only 8% of total stream length, the consequences could be devastating to aquatic communities if the diversity of these species pools are shaped by the geophysical diversity of stream types. Furthermore, our estimates of the extent of disturbance impacts are likely an underestimate of cumulative disturbance, as the extent of historical disturbances (e.g., mill ponds) is not represented in current maps of current land cover and infrastructures [83].

Stream classes showed strong associations with anthropogenic disturbance regimes. In some ways, this is not surprising as it is difficult to separate certain anthropogenic disturbances (e.g., agricultural land use) from natural geophysical types (e.g., valleys) [84]. In other cases, however, class-disturbance associations could reflect our inability to separate natural variation from human impacts on the landscape. For example, confinement was highly associated with impoundment and agriculture. Unlike other classes, confinement reflects the current condition (not reference condition) of valley geomorphology in the landscape based on recent DEM data; thus, we have no way to separate natural variation from human disturbance. In other cases, however, associations between classes and anthropogenic disturbances is related to increased propensity of some systems to face anthropogenic pressures. An unfortunate finding was that rare streams tended to have a larger portion of their membership disturbed (Fig 9). One probable reason is that large river systems are rare, yet face a higher likelihood of disturbance from dam regulation, impoundment, and cumulative upstream landscape disturbances. Additionally, despite highly heterogenous types of streams, humans tend to value relatively homogenous river types (e.g. stable and perennial flows, moderate sized supportive of recreation) [85]. This suggests that society may undervalue rare streams leading to higher prevalence of disturbance and lack of protection for these stream types.

Our conservation protection, restoration, and biological monitoring prioritization efforts yielded a cumulative total of 1328 classes that represented 87% of the physical diversity of streams, while only constituting 5% of total stream length in the Eastern US. While 77,000 km of streams seems like a large number and impractical for management, this estimate is quite reasonable given current stream management efforts. For instance, our restoration prioritization isolated 32,186 km of stream reaches or 2.1% of stream length in the Eastern US. In comparison, the documented national cumulative stream length falling within restoration projects as of 2005 was > 21,000 km [86]. Our restoration prioritization translates to 1463 km of streams per state in the Eastern US, which is quite higher than the average number of restored stream miles documented for Southeastern states, 169 km per state [87]. Our biological monitoring prioritization identifies 1% of streams, representing 24% of physical stream diversity in the Eastern US, which corresponds well to estimates of existing systematic aquatic biological surveys for the entire US (i.e., 5% of the nation’s area) [29].

An important caveat is that the prioritization scenarios we provide represent only a few examples of a wide range of possible scenarios that could be used to target sites for restoration, conservation, and monitoring. Furthermore, stream reaches identified in our assessment require field verification and more precise identification of impacts and needed restoration prescriptions. Likewise, our biological monitoring assessment was based on available open-access biodiversity data and may not reflect all sample occurrences. More importantly, our uncertainty assessment suggests that on a reach-by-reach basis, typologies could shift dramatically. Thus, care should be taken when interpreting our findings and more subsequent research is needed to ensure any prioritizations account for uncertainty.

### Utility of stream classifications, a case study

Layered stream classifications that represent the multidimensional characteristics of fluvial habitats can be used for many purposes. As our analysis showed, stream classifications can be used as an inventory of common-to-rare physical types that support conservation prioritization efforts; however, there are many other examples of potential applications. For example, stream classifications consolidate rich habitat information into typologies and hence, could be used for aquatic species distribution modeling (SDM). Layers of stream classes could be incorporated as variables in SDMs or, in cases of limited occurrence data, habitat typologies can be used as an “envelope” of habitat suitability [88].

Multiple frameworks suggest using stream classes as a template to examine disturbance-ecology relationships [18, 89]. Specifically, stream classes could be used to understand how different stream environments respond to disturbance, or even further, understand how physical properties mediate disturbances on ecological communities. Stream classes can be used to stratify stress-ecosystem response curves [90], which provide a mechanistic understanding of how disturbance alters habitat, which subsequently, structures ecological communities. For example, hydrologic classes have been used to stratify hydrologic [91] and ecological [92] responses to dam regulation.

Stream classes also offer a framework to guide restoration actions in two main ways. First, stream classes provide a multi-dimensional template to preliminarily identify reference streams that share similar physical, and presumably, ecological properties to disturbed streams in the process of restoration. Disturbance layers provide additional information to select sites meeting reference condition criteria or sites with similar disturbance regimes. Secondly, stream classes, if based on natural variation (as in our classification), represent an idealized reference condition. Class membership can be used to infer a range of expected conditions for streams in the absence of disturbance [91] and infer mitigation needs.

#### Case study

**Fig 12:**
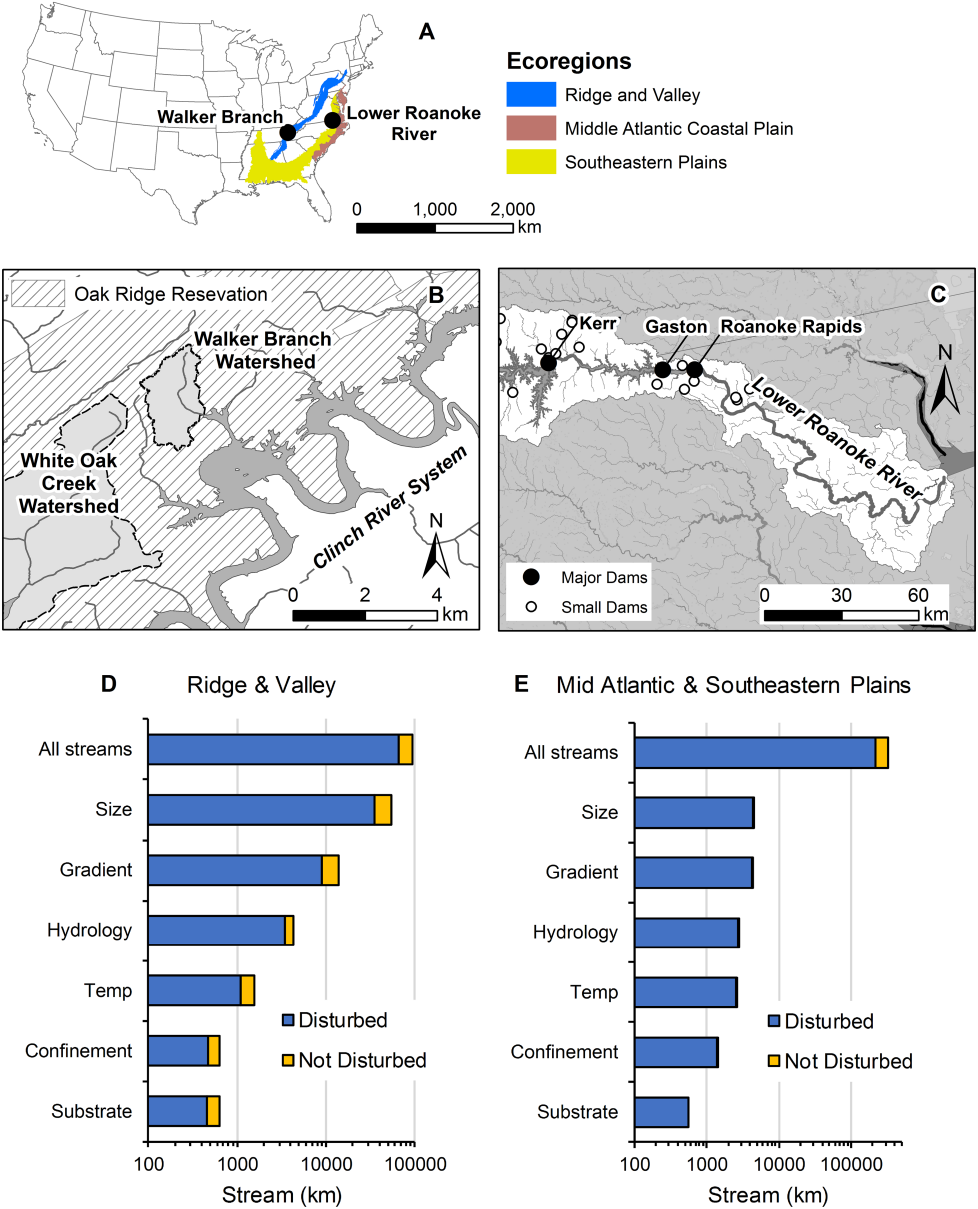
Case study examining streams similar to Walker Branch, Tennessee, and the Lower Roanoke River, North Carolina. (A) Locations of Walker Branch and the Lower Roanoke River and associated Ecoregions. Study side maps of (B) Walker Branch watershed and (C) the Lower Roanoke River. Streams in the (D) Ridge and Valley and (E) Mid-Atlantic and Southeastern Plains were sequentially filtered based on whether they matched the same typology as Walker Branch and the Lower Roanoke River, respectively. Sequential filtering started with size classes, and then was followed by gradient, hydrology, temperature, confinement, and substrate (i.e., in order of hypothesized importance in structuring stream ecosystems). Total length of streams matching filters is provided.

As an example of the utility of the stream classification, we provide a case study examining stream systems similar to Walker Branch, Tennessee, and the Lower Roanoke River, North Carolina. Walker Branch drains a relatively undisturbed and forested watershed in the Ridge and Valley Ecoregion (Fig 12) and has been the focus of almost 30 years of long-term stream ecology research on organic matter processing [93], nutrient cycling [94,95], ecosystem metabolism studies [96], and water chemistry dynamics [97]. As with other long-term ecological research, Walker Branch was selected as a representative of many streams with similar climate and landscapes across the US in order to extend biogeochemical research findings as broadly as possible [97]. Our layered classification approach provides a template to test this assumption. We incrementally selected streams in the Ridge and Valley Ecoregion and the entire Eastern US matching the layer typologies of Walker Branch, starting with size, followed by gradient, hydrology, temperature, confinement, and substrate (i.e., in order of hypothesized importance in structuring stream ecosystems). Based on our complex stream typologies, Walker Branch is a headwater, moderate-gradient, stable-high baseflow, cool, valley-confined, large-boulder type system. This unique typology represents less than 1% of streams (631 km) in the Ridge and Valley Ecoregion and less than 0.5% of streams (~4900 km) in the entire Eastern US (Fig 12, S2 Fig). Even if we use the simplified typology (i.e., removes substrate), these percentages remain the same. Additionally, the above percentages drop by 30–70% if only streams with minimal disturbances are considered (Fig 12). Although headwater systems represent > 50% of all stream length in the Eastern US, just the unique combination of headwater and moderate-gradient types is found in only 9% of all streams in the Eastern US. Depending on the research application, one or multiple layers can be selected to identify streams that match the physical properties under consideration to extend research findings to other systems.

Similar to Walker Branch, the Lower Roanoke River (LRR) has been an active location of research, but for different reasons, specifically the conservational importance of the river, its floodplain, and entry into Albermarle Sound in providing habitat for a large diversity of sensitive animal and plant species [98, 99, 100]. The majority of the mainstem Roanoke River and its tributaries are highly regulated by impoundments, the most downstream of which is Roanoke Rapids Dam (Fig 12). Below Roanoke Rapids Dam, the LRR flows freely for 326 km through the Southeastern Plains (SP) and eventually Middle Atlantic Coastal Plains (MACP) Ecoregions before entering the estuary (Fig 12). Because the Roanoke River has been a focus of intense management, primarily developing environmental flows [98, 100) and preserving natural floodplain inundation [99], identifying case studies or reference streams to guide management could be important. The predominant typology of the LRR is a large-river, very-low gradient, stable-high baseflow, warm, unconfined valley, small-boulder and coarse-gravel type system. Because big river systems are rare, we included both large and great river classes, which collectively only represent roughly 1% of all streams in both the SP and MACP Ecoregions, as well as the entire Eastern US (Fig 12). Considering only simplified typologies, less than 1400 km (0.4%) and less than 1900 km (0.1%) of streams share similar typologies to that of the LRR in the selected Ecoregions and entire Eastern US, respectively (Fig 12, S3 Fig). Virtually, all these systems (e.g., Pee Dee, Ocmulgee, Appalachicola River) are highly regulated by dams and could serve as ideal case studies (SI 8); however, the prospect of a minimally disturbed reference system with very similar properties to the LRR is unlikely.

### Classifications at stream-reach resolutions

Historically, stream classification efforts have remained divergent from the mapping ofstream-reaches, as most past efforts have typically been the focus of clustering discrete objects, as opposed to developing spatially comprehensive and contiguous characterizations of stream landscapes (except see [11]). Prior to the availability of large stream-reach datasets, many stream classifications remained conceptual frameworks that expanded our understanding of stream function and organization [30,82], but did not translate into widely-applicable and spatially coherent classification products. Ultimately, this limits the questions we can ask regarding the diversity of stream ecosystems within a large region. Our analysis suggests that stream physical diversity is very high and that similar stream types are patchily distributed in the landscape. The distribution of stream types tends to represent a balance between longitudinal forces of organization [38] versus patchy geomorphic controls [101]. For instance, stream types in some layers show strong patterns of regionality and longitudinal gradients (e.g., hydrology, temperature) whereas others occur as more discrete patches (e.g., gradient, confinement). However, combining all six layers results in highly variant stream types occurring within close proximity, which tends to agree with the concept that streams are organized as highly variable hydrogeomorphic patches [102].

Our stream classification and assessment of physical diversity should be viewed within the context of reach-scale segments (i.e., 1:100K scale or 1 km segments). We considered six different physical layers that could be characterized from available remote sensing products, many of which had resolutions > 30 meters. This approach will exclude measures of physical geomorphic diversity measured at the meso- or micro-habitat scales, i.e. 1 to 100 meters [30], such as within-channel geomorphology. In comparison, multiple studies have evaluated the heterogeneity of spatial variation in morphological units, bedforms, bars, and stream bed topography [103–106]. These studies generally conclude that geomorphological diversity is high, but the degree of heterogeneity is completely dependent upon the scale of analysis. Hence, combining our classification with those developed a meso- and micro-habitat scales would likely yield an exorbitant number of possible stream reach-meso-mico typologies across the Eastern US.

### Limitations and conclusions

Our estimation of riverscape diversity for the Eastern US is certainly an artifact of our approach and selection of layers. We fully recognize that there are multiple other physical and non-physical habitat layers that could have been included that would have increased the dimensionality of our stream diversity estimates. For instance, water chemistry, such as alkalinity, has been modeled for large regions of the US [107] and has been included in other stream classifications [23]. Additionally, other reach-scale layers depicting stream network configurations, such as tributary junctions, could have also been included to represent spatial arrangements of dendritic river systems. Tributary junctions create discontinuities among stream patches [108] and induce large effects on the ecosystem processes and habitat diversity of rivers they enter [36]. However, the number of stream typologies in our analysis was already quite large for informing practical approaches to conservation and management. Moreover, we found that combinations of each subsequent layer beyond five habitat attributes did not yield large increases in our diversity score. Furthermore, the cumulative error propagation from the addition of more layers would likely lead to more instability in stream typologies.

Another difficult, but necessary, aspect of developing classifications is separating multi-dimensional data into distinct groups. Realistically, however, many environmental phenomena likely represent gradients in values as opposed to distinct thresholds or boundaries. Subsequently, observations with environmental values near the fringe of cluster’s multidimensional space could theoretically share membership with multiple different clusters. Furthermore, probabilities of “fuzzy membership” for each classification is likely compounded with the assemblage of multiple habitat layers. Hence, layered approaches to stream classifications could overestimate the physical diversity of streams because all variation is not jointly consolidated through clustering. However, individual stream types, and subsequently, measures of stream diversity, may be difficult to interpret from agglomerative clustering solutions. Alternatively, preserving the identity of habitat layers within final stream typologies is important to understanding and communicating the functional components of streams [36].

Extrapolating habitat conditions to a large number of unsampled stream reaches based on empirically-driven predictive models does not come without limitations. For example, this approach requires sufficient sample sizes of empirical observations to model habitat conditions in unsampled streams. However, this requirement comes at a cost to limitations in our choice of habitat layers and associated classes. For example, a more sophisticated classification of thermal regimes [e.g., 21] would have been ideal, as opposed to relying on a more simplified classification of July-August averages; however, sample sizes from Maheu et al.’s [21] classification (n = 50 in Eastern US) were not sufficient to support model development. Additionally, our modeled substrate values are biased towards larger substrate classes because we used a weighted mean diameter of particles, as this could be readily calculated from empirical data sources.

Such approaches will also induce uncertainty into estimates of habitat conditions in unsampled stream reaches; thus, any interpretation of stream typologies should take our estimates of model error into consideration. After accounting for uncertainty in classes, we observed little variation in our diversity and rarity estimates and their overlap with disturbance regimes. However, on a reach-by-reach basis, uncertainty in class membership creates a high potential for shifts in typologies. A common observation is that empirical observation networks within stream environments are under-representative of the diversity of stream ecosystems [109]. While we acknowledge that our models are not immune to the issues arising from data limitations, our compilations of empirical observations are not small (i.e., 500–2000 samples) and represent >98% of the overall variation of stream landscape attributes for the entire region.

The resolution of our classification and related measures of diversity are completely dependent upon the scale of the underlying datasets used in its development (i.e., NHDPlus V1). For instance, our classification omits very small headwaters and ephemeral streams and hence, underestimates the diversity of stream types. Likewise, assessments of natural variation and anthropogenic disturbances are also dependent upon available information, which may be inaccurate. In particular, estimates of fragmentation by dams are dependent upon data that appropriately characterize barriers. However, these datasets are many times incomplete and miss small impoundments [110]. While our assessment excluded information at finer resolutions, our classification approach does not preclude incorporating higher-resolution layers (e.g. tributaries, pools, riffles) as patches within reaches.

Wohl [85] concludes that most studies evaluating heterogeneity in stream geomorphology suggest that diversity of stream environments high. While our focus is on the reach or segment scale rather than within-reach scale, our findings concurrently suggest that stream physical diversity is extremely high. By extrapolating rates of diversity from our classification to the entire conterminous US (1 million:2.6 million reaches), we estimate there could be anywhere from 4,000 to 14,5000 different types of stream reaches present. Understanding this level of diversity in important for representing these ecosystems in Earth System models, but also potentially useful to practical applications, such as prioritizing streams for conservation, restoration, and monitoring.

## Supporting information

S1 Fig Drainage-area thresholds supporting size classes. Breaks or threshold values found in the literature for stream size classifications based on upstream drainage area.
(TIF)

S2 Fig Walker Branch case study sites. Stream reaches in the Ridge and Valley Ecoregion scored according to their similarity to Walker Branch.
(PDF)

S3 Fig Roanoke River case study sites. Stream reaches in the Middle Atlantic Coastal Plain and Southeastern Plains scored according to their similarity to the Lower Roanoke River.
(PDF)

S1 Table Stream typology examples. Examples of the 30 most dominant and 30 rarest simplified stream typologies in the eastern US (does not include substrate classes). Codes for classes are provided in Table 1. All typologies for both simple and complex approaches are provided in SI.
(PDF)

S2 Table Disturbance patterns and uncertainty in typologies. Comparison of disturbance patterns between the original simple typology and typology scenarios arising from class uncertainty. The % physical diversity is calculated relative to the number of typologies with lengths > 1km whereas the % stream length is calculated relative to the total length of all streams in the Eastern US.
(PDF)

S1 File Predictor variables and model performance. Spatial variables, their sources, and whether they were used to model hydrologic classes, temperature classes, substrate size or bankfull width. Results of random forest models are provided including performance, variable importance, and the confusion matrix (hydrologic classes).
(XLSX)

S2 File Observation data representation. Representativeness of the subset of streams containing empirical observations of hydrology, temperature, and substrate on the overall variation represented by all streams in the region.
(PDF)

S3 File Drainage-area vs. flow relationships. Relationships between drainage area and mean annual flow stratified by different climate zones and hydrologic regions for the Eastern US.
(PDF)

S4 File Methods for quantifying typology uncertainty. Approach and results of examining the effect of uncertainty of mapping classes to stream reaches on stream typologies and rarity estimates.
(PDF)

S5 File Simple and complex stream typologies. List of simple (substrate excluded) and complex (all habitat layers) stream typologies found in the analysis and total length occupied in streams in the eastern US. Stream typologies for all uncertainty scenarios and their length are also provided.
(XLSX)
