# Supplementary figures and images for: A stream classification system to explore the physical habitat diversity and anthropogenic impacts in riverscapes of the eastern United States
Source: PLoS One. 2018 Jun 20;13(6):e0198439. doi: 10.1371/journal.pone.0198439 (PMC6010261; doi:10.1371/journal.pone.0198439)

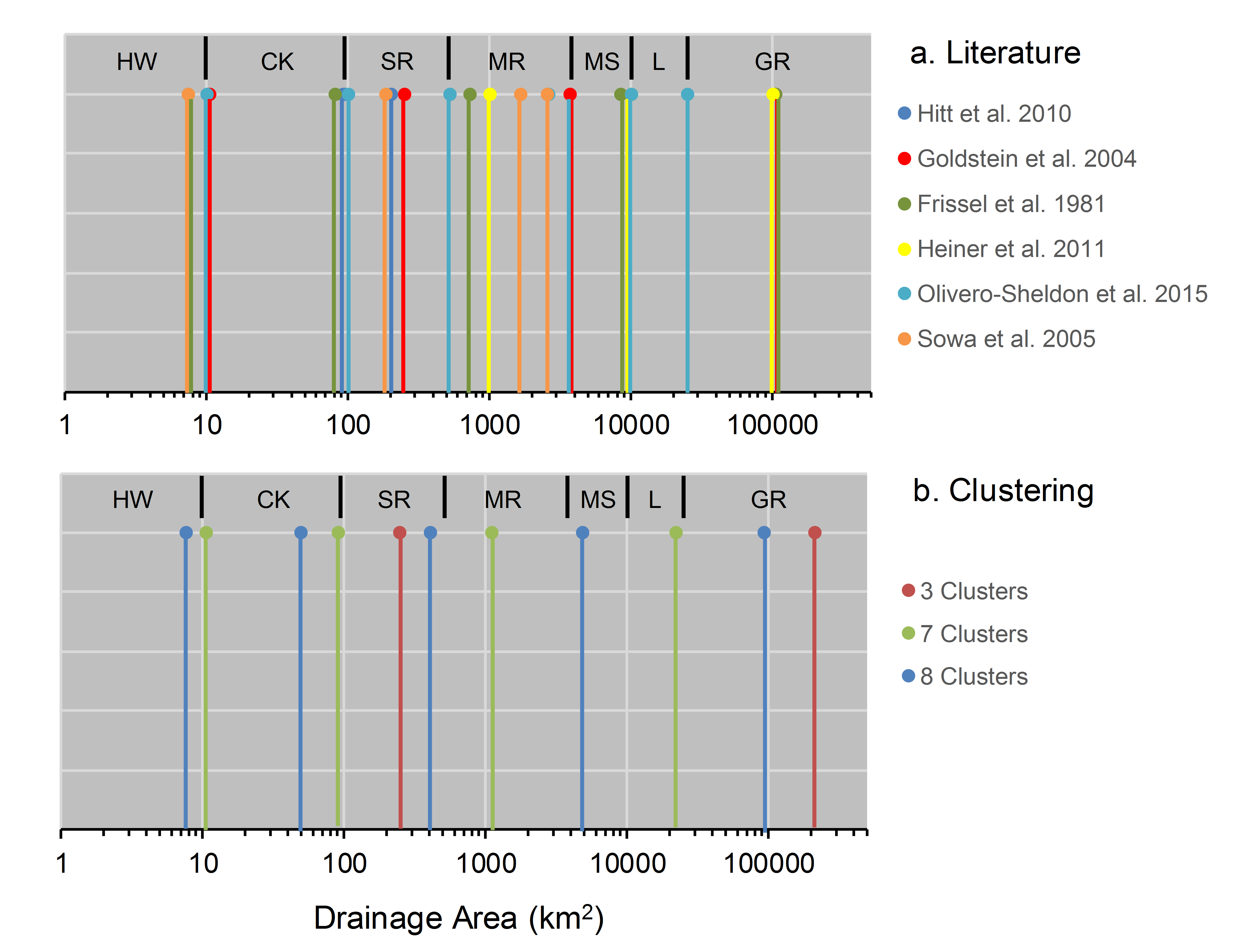

Supplement: S1 Fig — Breaks or threshold values found in the literature for stream size classifications based on upstream drainage area. (TIF) [file pone.0198439.s001.tif]
