# Supplementary material for: A stream classification system to explore the physical habitat diversity and anthropogenic impacts in riverscapes of the eastern United States
Source: PLoS One. 2018 Jun 20;13(6):e0198439. doi: 10.1371/journal.pone.0198439 (PMC6010261; doi:10.1371/journal.pone.0198439)
Supplement: S2 Fig — Stream reaches in the Ridge and Valley Ecoregion scored according to their similarity to Walker Branch. (PDF) [file pone.0198439.s002.pdf]

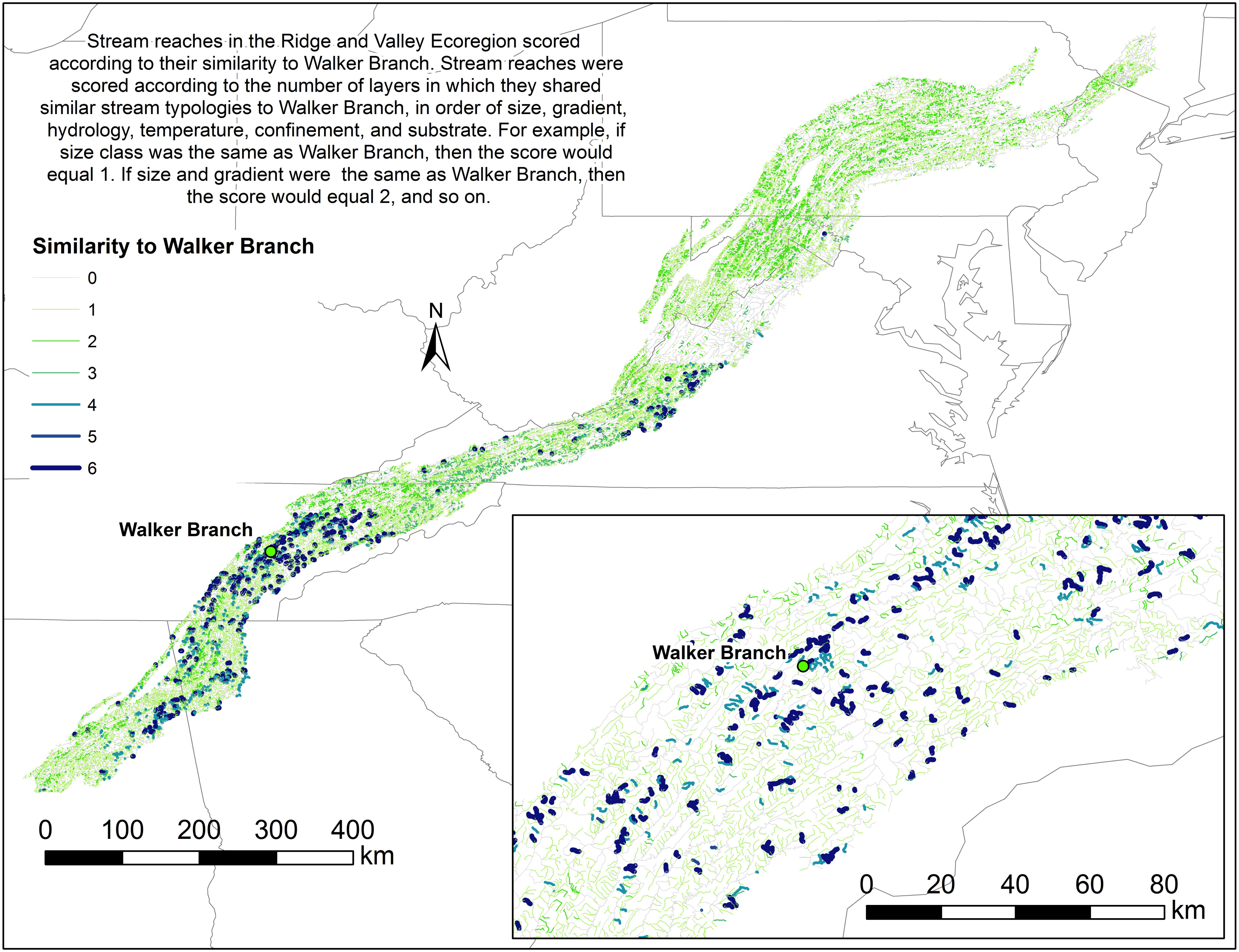

Stream reaches in the Ridge and Valley Ecoregion scored according to their similarity to Walker Branch. Stream reaches were scored according to the number of layers in which they shared similar stream typologies to Walker Branch, in order of size, gradient, hydrology, temperature, confinement, and substrate. For example, if size class was the same as Walker Branch, then the score would equal 1. If size and gradient were the same as Walker Branch, then the score would equal 2, and so on.
Similarity to Walker Branch
0
1
2
3
4
5
6
N
Walker Branch
0
100
200
300
400
km
Walker Branch
0
20
40
60
80
km
