# Supplementary material for: A stream classification system to explore the physical habitat diversity and anthropogenic impacts in riverscapes of the eastern United States
Source: PLoS One. 2018 Jun 20;13(6):e0198439. doi: 10.1371/journal.pone.0198439 (PMC6010261; doi:10.1371/journal.pone.0198439)
Supplement: S3 Fig — Stream reaches in the Middle Atlantic Coastal Plain and Southeastern Plains scored according to their similarity to the Lower Roanoke River. (PDF) [file pone.0198439.s003.pdf]

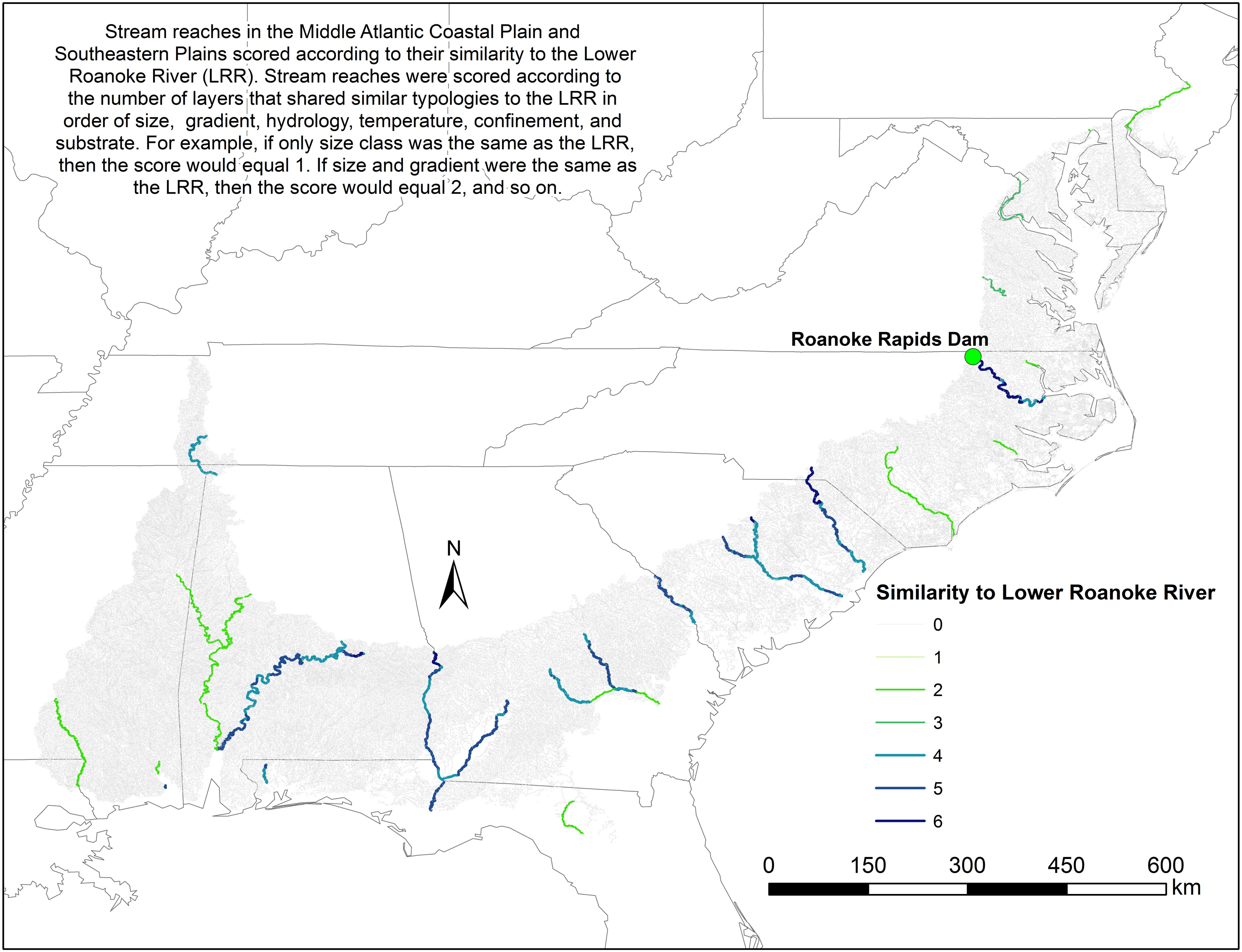
Stream reaches in the Middle Atlantic Coastal Plain and Southeastern Plains scored according to their similarity to the Lower Roanoke River (LRR). Stream reaches were scored according to the number of layers that shared similar typologies to the LRR in order of size, gradient, hydrology, temperature, confinement, and substrate. For example, if only size class was the same as the LRR, then the score would equal 1. If size and gradient were the same as the LRR, then the score would equal 2, and so on.
Roanoke Rapids Dam
N
Similarity to Lower Roanoke River
0
1
2
3
4
5
6
0
150
300
450
600
km
