# Supplementary material for: A stream classification system to explore the physical habitat diversity and anthropogenic impacts in riverscapes of the eastern United States
Source: PLoS One. 2018 Jun 20;13(6):e0198439. doi: 10.1371/journal.pone.0198439 (PMC6010261; doi:10.1371/journal.pone.0198439)
Supplement: S1 Table — Examples of the 30 most dominant and 30 rarest simplified stream typologies in the eastern US (does not include substrate classes). Codes for classes are provided in Table 1. All typologies for both simple and complex approaches are provided in SI. (PDF) [file pone.0198439.s004.pdf]

S1 Table. Stream Typology Examples

Examples of the 30 most dominant and 30 rarest simplified stream typologies in the eastern US (does not include substrate classes). Codes for classes are provided in Table 1. All typologies for both simple and complex approaches are provided in SI.

Most Dominant		Rarest	
**Typology**	**Length (km)**	**Typology**	**Length (km)**
HW-M-SHB-CL-UC	47540	SR-H-SHB-CW-C	1
HW-M-PR1-CL-UC	41526	GR-L-SHB-CW-C	1.003
HW-M-PR1-CW-UC	41089	GR-MH-PR2-CC-UC	1.008
HW-L-PR1-CW-UC	25897	SR-L-LTR-CW-C	1.019
HW-M-PR2-CD-UC	25354	CK-MH-SSG-CC-UC	1.021
CK-L-PR1-CW-UC	23798	MR-M-UI-CW-C	1.028
HW-M-SHB-CW-UC	19220	SR-H-PR1-CW-UC	1.031
CK-L-SHB-CW-UC	17809	HW-L-PR2-CW-C	1.039
HW-H-PR2-CD-C	16877	SR-H-SHB-CL-UC	1.042
HW-MH-PR2-CD-UC	16833	MR-H-PR2-CC-MC	1.043
HW-L-PR1-CC-UC	15968	HW-MH-PR1-W-MC	1.043
HW-M-PR1-CC-UC	15501	LR-L-PR1-CL-MC	1.059
CK-M-PR2-CD-UC	14389	MS-MH-PR2-CC-C	1.059
CK-L-PR1-CC-UC	14271	SR-MH-PR1-CL-MC	1.064
HW-M-PR1-CL-MC	13216	LR-MH-SHB-CC-UC	1.077
HW-M-SHB-CL-MC	12802	HW-L-IF2-W-C	1.089
HW-M-PR2-CC-UC	12338	MR-MH-PR1-CW-C	1.091
HW-L-UI-CC-UC	12047	SR-MH-SM2-CD-C	1.101
HW-M-UI-CL-UC	12042	HW-H-LTR-CW-C	1.104
CK-L-PR2-CC-UC	12006	GR-L-PR1-W-MC	1.106
CK-L-PR1-CL-UC	12003	MS-H-PR2-CD-C	1.11
HW-M-UI-CC-UC	11909	SR-M-SSG-CW-C	1.112
HW-MH-PR1-CC-C	11703	SR-VL-Unc-Unc-C	1.126
HW-MH-PR2-CD-MC	11515	HW-L-PR1-CD-C	1.133
CK-M-PR2-CC-UC	11251	MS-H-PR1-CW-UC	1.139
HW-M-PR1-CL-C	10725	SR-MH-PR1-CC-UC	1.14
HW-H-PR2-CD-MC	10175	MR-VH-SHB-CL-C	1.162
HW-L-SHB-CW-UC	9939	MR-MH-PR2-CL-UC	1.168
