# Supplementary material for: A stream classification system to explore the physical habitat diversity and anthropogenic impacts in riverscapes of the eastern United States
Source: PLoS One. 2018 Jun 20;13(6):e0198439. doi: 10.1371/journal.pone.0198439 (PMC6010261; doi:10.1371/journal.pone.0198439)
Supplement: S2 Table — Comparison of disturbance patterns between the original simple typology and typology scenarios arising from class uncertainty. The % physical diversity is calculated relative to the number of typologies with lengths > 1km whereas the % stream length is calculated relative to the total length of all streams in the Eastern US. (PDF) [file pone.0198439.s005.pdf]

S2 Table. Disturbance Patterns and Uncertainty in Typologies

Comparison of disturbance patterns between the original simple typology and typology scenarios arising from class uncertainty. The % physical diversity is calculated relative to the number of typologies with lengths > 1km whereas the % stream length is calculated relative to the total length of all streams in the Eastern US.

Typology Scenario	Criteria	Number Classes	Stream Length (km)	% Physical Diversity	% stream length
**Simple**	Class Disturbance = 100%	306	136521	20.1	9.07
	Disturbance > 95%	492	250888	32.3	16.7
	100% Impounded	36	252	2.37	0.02
**Size**	Class Disturbance = 100%	311	136850	20.5	9.10
	Disturbance > 95%	503	250818	33.2	16.7
	100% Impounded	35	250	2.31	0.02
**Gradient**	Class Disturbance = 100%	315	136753	20.6	9.09
	Disturbance > 95%	499	245850	32.6	16.3
	100% Impounded	37	293	2.42	0.02
**Hydrology**	Class Disturbance = 100%	309	135835	18.7	9.03
	Disturbance > 95%	527	247468	31.9	16.4
	100% Impounded	31	260	1.88	0.02
**Temperature**	Class Disturbance = 100%	267	136055	18.6	9.04
	Disturbance > 95%	440	237794	30.7	15.8
	100% Impounded	16	201	1.12	0.01
**Confinement**	Class Disturbance = 100%	301	136508	20.0	9.07
	Disturbance > 95%	493	250703	32.7	16.7
	100% Impounded	36	252	2.39	0.02
**All layers**	Class Disturbance = 100%	382	138287	18.8	9.19
	Disturbance > 95%	552	159857	27.2	10.6
	100% Impounded	6	25	0.30	<0.01
