# Supplementary material for: A stream classification system to explore the physical habitat diversity and anthropogenic impacts in riverscapes of the eastern United States
Source: PLoS One. 2018 Jun 20;13(6):e0198439. doi: 10.1371/journal.pone.0198439 (PMC6010261; doi:10.1371/journal.pone.0198439)
Supplement: S2 File — Representativeness of the subset of streams containing empirical observations of hydrology, temperature, and substrate on the overall variation represented by all streams in the region. (PDF) [file pone.0198439.s007.pdf]

S2 File.

Observation Data Representation

Representativeness of the subset of streams containing empirical observations of hydrology, temperature, and substrate on the overall variation represented by all streams in the region.

A Stream Classification System to Explore the Physical Habitat Diversity and Anthropogenic Impacts in Riverscapes of the Eastern United States

Ryan A. McManamay, Matthew J. Troia, Christopher R. DeRolph, Arlene Olivero Sheldon, Analie Barnett, Shih-Chieh Kao, Mark Anderson

Because the field observations represented only a subset of streams, we questioned whether our observation datasets were representative of the overall variation of streams within the Eastern US. Of the 200 network-accumulated variables (SI 1), we isolated 114 variables that were common among all models predicting hydrology, temperature, and substrate conditions. We conducted a Principal Components Analysis (PCA) on all 114 variables summarized for all stream reaches in the region and determined the variation explained by each PC. Using the PCs explaining 95% of the total variation, we then calculated the proportion of stream reaches with PC scores falling outside the range (min, max) of PC scores represented by each of our observation datasets. These proportions were then multiplied by the variation explained by that PC. The total variation not accounted for by our observed datasets was then calculated by summing the proportioned variation across all PCs.

The first 75 PCs explained 95% of the variation in the 114 variables for all streams in the region. On average (across all PCs), less than 1% of streams for the Eastern US fell outside of the range of PC scores represented by our observation datasets (maximum=3.5%). When examining the cumulative proportion of variation of all streams in the region, our observation datasets capture over 98.5% of the overall variation represented by the entire stream dataset (Figure S2 1).

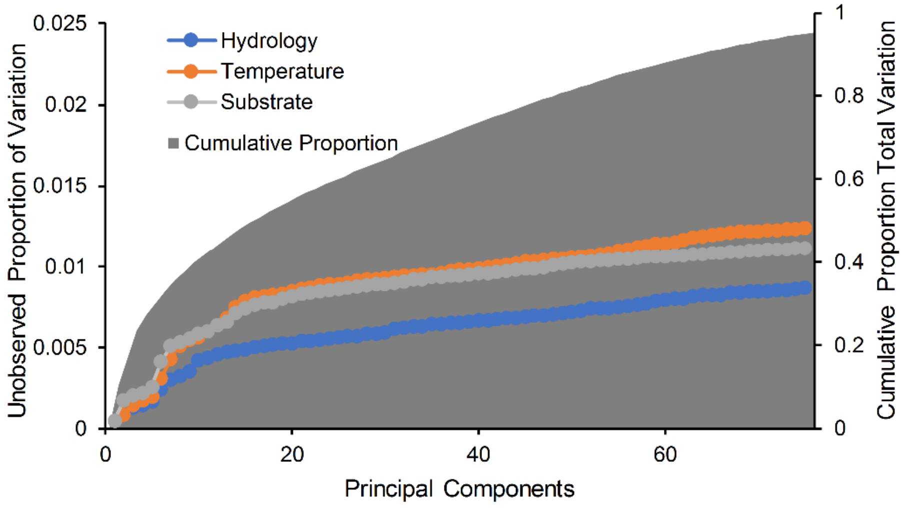

Figure 1. Unobserved proportion of total variation not accounted for by streams containing hydrologic, temperature, or substrate observations (left y-axis). Cumulative proportion of total variation comprised by all streams in the Eastern US (right y-axis).
