# Supplementary material for: A stream classification system to explore the physical habitat diversity and anthropogenic impacts in riverscapes of the eastern United States
Source: PLoS One. 2018 Jun 20;13(6):e0198439. doi: 10.1371/journal.pone.0198439 (PMC6010261; doi:10.1371/journal.pone.0198439)
Supplement: S3 File — Relationships between drainage area and mean annual flow stratified by different climate zones and hydrologic regions for the Eastern US. (PDF) [file pone.0198439.s008.pdf]

S3 File

Drainage-Area vs. Flow Relationships

Relationships between drainage area and mean annual flow stratified by different climate zones and hydrologic regions for the Eastern US.

A Stream Classification System to Explore the Physical Habitat Diversity and Anthropogenic Impacts in Riverscapes of the Eastern United States

Ryan A. McManamay, Matthew J. Troia, Christopher R. DeRolph, Arlene Olivero Sheldon, Analie Barnett, Shih-Chieh Kao, Mark Anderson

Relationships were developed using NHDplus V1 estimates of mean annual flow and upstream drainage area. Table 1 evaluates relationships between mean annual discharge and size correlates for 9 gridded blocks whereas Table 2 shows the same information, but according to 2-digit hydrologic regions.

Table 1. Relationships between mean annual discharge and drainage area, and mean annual discharge and Strahler order among nine gridded blocks in the eastern US. Latitudinal grid breaks are as follows: South <32.7°N, Mid 32.7–40.3°N, North > 40.3°N; Longitudinal grid breaks are as follows: East <75.9°W, Central 75.9–84.8°W, West >84.8°W.

Stream size correlate *	Grid cell	R^2	y-intercept	slope
Strahler order ~ Mean annual discharge	North-west	0.59	1.25	0.96
	North-central	0.67	1.23	1.08
	North-east	0.65	1.02	1.02
	Mid-east	0.70	1.19	1.13
	Mid-central	0.59	1.38	1.00
	Mid-east	0.46	1.46	0.72
	South-west	0.63	1.28	1.09
	South-central	0.41	1.62	0.71
	South-east	n/a	n/a	n/a
Drainage area ~ Mean annual discharge	North-west	0.99	0.46	1.00
	North-central	0.98	0.36	0.98
	North-east	0.99	0.17	0.99
	Mid-east	0.99	0.26	0.99
	Mid-central	0.99	0.33	0.98
	Mid-east	1.00	0.29	0.99
	South-west	0.99	0.23	0.99
	South-central	0.99	0.46	0.99
	South-east	n/a	n/a	n/a

* Mean annual discharge and drainage area were $\log_{10}$-transformed prior to linear model fitting.

Table 2. Relationships between mean annual discharge and drainage area, and mean annual discharge and Strahler order among 2-digit hydrologic regions of the eastern US.

Stream size correlate *	HUC2	R^2	y-intercept	slope
Strahler order ~ Mean annual discharge	1	0.67	1.01	1.03
	2	0.64	1.26	0.99
	3	0.51	1.48	0.91
	4	0.60	1.27	0.99
	5	0.76	1.11	1.18
	6	0.71	1.13	1.16
	7	0.04	1.11	0.12
	8	0.71	1.36	0.95
Drainage area ~ Mean annual discharge	1	1.00	0.17	0.99
	2	0.99	0.30	0.98
	3	0.99	0.33	0.98
	4	0.98	0.42	0.98
	5	0.99	0.31	0.99
	6	0.99	0.16	0.99
	7	0.99	0.44	1.01
	8	1.00	0.26	1.00

* Mean annual discharge and drainage area were $\log_{10}$-transformed prior to linear model fitting.
