# Supplementary material for: A stream classification system to explore the physical habitat diversity and anthropogenic impacts in riverscapes of the eastern United States
Source: PLoS One. 2018 Jun 20;13(6):e0198439. doi: 10.1371/journal.pone.0198439 (PMC6010261; doi:10.1371/journal.pone.0198439)
Supplement: S4 File — Approach and results of examining the effect of uncertainty of mapping classes to stream reaches on stream typologies and rarity estimates. (PDF) [file pone.0198439.s009.pdf]

S4 File

Methods for Quantifying Typology Uncertainty

Examining the effect of uncertainty of mapping classes to stream reaches on stream typologies and rarity.

A Stream Classification System to Explore the Physical Habitat Diversity and Anthropogenic Impacts in Riverscapes of the Eastern United States

Ryan A. McManamay, Matthew J. Troia, Christopher R. DeRolph, Arlene Olivero Sheldon, Analie Barnett, Shih-Chieh Kao, Mark Anderson

We estimated uncertainty for each layer in different ways (Table SX 1). For size and gradient, we compared our drainage area (km^2) and slope estimates from NHDPlus V1 (derived from 30-m DEM) to values provided in NHDPlus V2 (derived from 30-m DEM). Hydrologic classes were probabilistically assigned to stream reaches using random forest models, which yielded probabilities of a given stream reach having shared membership across all possible hydrologic classes. Predicted temperature and substrate values were compared to observed values to yield deviation estimates. Uncertainty in confinement was determined by measuring deviation in predicted bankfull width values compared to observed values.

Table S5 1. Sources of uncertainty used to calculate deviation values for re-assigning class membership.

Layer	Source of Uncertainty	% Deviation
Size	Comparison of drainage area (km^2) from NHDPlus V1 to NHDPlus V2	2.34
Gradient	Comparison of slope values NHDPlus V1 and NHDPlus V2	13.8
Hydrology	Probabilities of class assignment from random forests	Varies
Temperature	Deviation of observed vs. predicted values (°C)	3.86
Confinement	Variation in bankfull width area (km^2) compared to valley floodplain	10.0
Substrate	Deviation of observed vs. predicted values (mm)	39.0

For all layers except hydrology, we calculated % deviation estimates. (Table SX 1). We then applied + and - deviation to all values for each layer to get low and high estimates for all stream reaches. Using these low/high values, we then re-classified types for each layer and stream reach based on class partition thresholds provided in Table 1 (main manuscript). If thresholds were exceeded, we determined a 2nd most probable class for each stream reach. If thresholds were not exceeded, the class remained

unchanged. In most cases, only the low or high (not both) values resulted in a class change. In cases where both values resulted in class changes, we selected the class with values closest to the nearest threshold. For hydrology, we assigned the 2nd most probable class as provided by random forest prediction, only if the probability of membership of the primary class was <0.50.

We developed a series of new stream typology scenarios (i.e. layer combinations) by varying each layer independently and then varying all layers collectively. We then compared the number, cumulative length, and rarity (<10th percentile) of each new typology scenario to the original simplified typologies (Table SX 2). We also determined the agreement between each scenario and the original simplified typologies by calculating the % stream mileage sharing similar typologies. Additional, we calculated the % of stream mileage where reaches were defined as rare in both the simplified typology and each scenario (Table SX 3).

Table S5 2. Comparison of the number of all and rare typologies of uncertainty scenarios in comparison to the original simplified typology.

	Simple	Variant Size	Variant Gradient	Variant Hydrology	Variant Temperature	Variant Confinement	All Layers Varied
Total Types	1983	1974	1987	2140	1872	1966	2679
Total Types >1km	1521	1514	1529	1650	1434	1506	2027
Total Length >1km	1379591	1379594	1379440	1379440	1379448	1379442	1379425
Rare Types >1km	1217	1210	1220	1216	988	1126	1501
Rare Length >1km	138401	138433	138299	90167	66191	90734	138206
% Rare Types >1km	80.0	79.9	79.8	73.7	68.9	74.8	74.1
Rare Types all	1679	1670	1678	1706	1427	1586	2154
Rare Length all	138526	138554	138425	90293	66310	90858	138347

Table S5 3. Agreement between the original simple typology

	% Agreement All Streams					
	Variant Size Class	Variant Gradient	Variant Hydrology	Variant Temperature	Variant Confinement	All Layers Varied
Simple Typologies	95.6	78.9	42.0	33.7	96.9	12.3
	% Agreement Rare Streams					
	Variant Size Class	Variant Gradient	Variant Hydrology	Variant Temperature	Variant Confinement	All Layers Varied
Simple Typologies	98.4	87.4	45.2	39.1	65.4	31.2
